# Differences in MWCNT- and SWCNT-induced DNA methylation alterations in association with the nuclear deposition

**DOI:** 10.1186/s12989-018-0244-6

**Published:** 2018-02-09

**Authors:** Deniz Öner, Manosij Ghosh, Hannelore Bové, Matthieu Moisse, Bram Boeckx, Radu C. Duca, Katrien Poels, Katrien Luyts, Eveline Putzeys, Kirsten Van Landuydt, Jeroen AJ Vanoirbeek, Marcel Ameloot, Diether Lambrechts, Lode Godderis, Peter HM Hoet

**Affiliations:** 1 Laboratory of Toxicology, Unit of Environment and Health, Department of Public Health and Primary Care, KU Leuven, 3000 Leuven, Belgium; 2 Centre for Surface Chemistry and Catalysis, KU Leuven, Celestijnenlaan 200F, 3001 Leuven, Belgium; 3 Biomedical Research Institute, Agoralaan Building C, Hasselt University, 3590 Diepenbeek, Belgium; 4 Laboratory for Translational Genetics, Department of Human Genetics, KU Leuven, 3000 Leuven, Belgium; 5 Laboratory for Translational Genetics, VIB Centre for Cancer Biology, VIB, 3000 Leuven, Belgium; 6 Laboratory for Occupational and Environmental Hygiene, Unit of Environment and Health, Department of Public Health and Primary Care, KU Leuven, 3000 Leuven, Belgium; 7 Department of Oral Health Sciences, Unit of Biomaterials (BIOMAT), KU Leuven, 3000 Leuven, Belgium; 8 External Service for Prevention and Protection at Work, IDEWE, B-3001, Leuven, Belgium

**Keywords:** Carbon nanotubes, DNA methylation, Gene expression, Nuclear uptake, Toxicity, Epigenetics, Epigenomics, Genotoxicity, Nanoparticles, Nanomaterials, In vitro

## Abstract

**Background:**

Subtle DNA methylation alterations mediated by carbon nanotubes (CNTs) exposure might contribute to pathogenesis and disease susceptibility. It is known that both multi-walled carbon nanotubes (MWCNTs) and single-walled carbon nanotubes (SWCNTs) interact with nucleus. Such, nuclear-CNT interaction may affect the DNA methylation effects.

In order to understand the epigenetic toxicity, in particular DNA methylation alterations, of SWCNTs and short MWCNTs, we performed global/genome-wide, gene-specific DNA methylation and RNA-expression analyses after exposing human bronchial epithelial cells (16HBE14o- cell line). In addition, the presence of CNTs on/in the cell nucleus was evaluated in a label-free way using femtosecond pulsed laser microscopy.

**Results:**

Generally, a higher number of SWCNTs, compared to MWCNTs, was deposited at both the cellular and nuclear level after exposure. Nonetheless, both CNT types were in physical contact with the nuclei. While particle type dependency was noticed for the identified genome-wide and gene-specific alterations, no global DNA methylation alteration on 5-methylcytosine (5-mC) sites was observed for both CNTs. After exposure to MWCNTs, 2398 genes were hypomethylated (at gene promoters), and after exposure to SWCNTs, 589 CpG sites (located on 501 genes) were either hypo- (*N* = 493 CpG sites) or hypermethylated (*N* = 96 CpG sites).

Cells exposed to MWCNTs exhibited a better correlation between gene promoter methylation and gene expression alterations. Differentially methylated and expressed genes induced changes (MWCNTs > SWCNTs) at different cellular pathways, such as p53 signalling, DNA damage repair and cell cycle. On the other hand, SWCNT exposure showed hypermethylation on functionally important genes, such as SKI proto-oncogene (*SKI*), glutathione S-transferase pi 1 (*GTSP1*) and shroom family member 2 (*SHROOM2*) and neurofibromatosis type I (*NF1*), which the latter is both hypermethylated and downregulated.

**Conclusion:**

After exposure to both types of CNTs, epigenetic alterations may contribute to toxic or repair response. Moreover, our results suggest that the observed differences in the epigenetic response depend on particle type and differential CNT-nucleus interactions.

**Electronic supplementary material:**

The online version of this article (10.1186/s12989-018-0244-6) contains supplementary material, which is available to authorized users.

## Background

Carbon nanotubes (CNTs) are a class of graphene-based engineered nanomaterials. These have a tubular and fibre structure with a diameter in nanometer (nm). CNTs can be divided into categories by the number of layers of the rolled-up graphene, which will define their diameter size: e.g. single-walled CNTs (SWCNTs) with a diameter between 0.7 and 3 nm and multi-walled CNTs (MWCNTs) with a diameter between 10 and 200 nm [1]. SWCNTs are present in stiff, rope-like bundles due to the increased van der Waals forces caused by their extremely small diameter and high surface area. In contrast, MWCNTs can be present in agglomerated, curly or needle-like structure.

Since CNTs display high electrical and thermal conductivity, mechanical durability and functionalization properties, they are valuable nanomaterials for the use in industry (e.g. surface films and coatings, microelectronics, energy storage, composites) and for biotechnological and biomedical approaches [2]. The market of CNTs is expected to keep on growing in the next five years based on several online market reports.

The increase in the production of CNTs raised concern regarding inhalation toxicity because of their fibre shape that is similar to asbestos fibres. In addition, their nano dimensions might result in unexpected toxic and adverse effects. For instance, It was found that MWCNTs are internalized in cells through both direct penetration and endocytosis [3]. Endosomal leakage has been seen since MWCNTs are able to pierce lysosomes because of their greater diameter, leaving fibres within the cytosol [3]. In particular, this effect might increase the inflammatory response and interaction of MWCNTs with cytosolic materials such as nucleus, proteins, organelles and RNAs. In contrast, bundled SWCNTs are not able to pierce the cell membranes due to their physicochemical properties (e.g. extremely small diameter, appearance in bundles caused by van der Waals forces) but are internalized through endocytosis [4].

Subtle DNA methylation or gene expression alterations mediated by CNT exposure might contribute to disease progression or susceptibility [5–8]. Noteworthy, in cancer cells, typically global hypomethylation occurs with gene-specific hypermethylation. Global hypomethylation (in particular, hypomethylation of centromeric regions), which leads to uncondensed DNA, might be linked with impaired segregation of the chromosomes and increased genotoxicity parameters, such as increased formation rate of micronucleus and chromosomal breaks [7]. In addition, gene expression alterations or mutations on *DNMT1* gene will lead to global hypomethylation after cell divisions (passive DNA demethylation). Gene-specific hypermethylation is linked with gene silencing (downregulation of gene expression). In addition, DNA methylation on cytosine residues ‘hot spots’ for spontaneous mutations due to impaired DNA repair mechanisms [9]. Gene-specific hypomethylation may upregulate gene expression, stress-response or pro-oncogenic signalling mechanisms of the cell.

For the case of CNTs, MWCNT-uptake in *Allium cepa* resulted in increase in global DNA methylation and genotoxic response Ghosh et al. [10]. In mammalian cells and in vivo, changes in DNA methylation after CNT-exposure occurred. After intra-tracheal administration of CNTs, the gene promoter region of ATM serine/tyrosine kinase (*ATM*), which functions in DNA damage pathway, was altered in lung tissue of mice Tabish et al. [11]. Gene-specific alterations in the inflammatory response genes [such as interferon-gamma (*IFN-γ*) and tumour necrosis factor alpha (*TNF-α*)] and global DNA hypomethylation in the lung and blood after exposure to MWCNTs were observed and correlated with cytokine production and collagen deposition Brown et al. [12]. It has also been reported that exposure to some carbon-based nanoparticles (carbon black, short MWCNTs and SWCNTs) cause DNA hypermethylation at the global level in adenocarcinoma human alveolar basal epithelial cells (A549 cell line) J. Li et al. [13]. Sierra et al., observed hypomethylation of 755 CpG sites in the human lung epithelial cells (BEAS-2B) exposed to MWCNTs and four weeks of exposure induce more differentially methylated probes than their two weeks of exposure counterparts Sierra et al. [14]. In a recent study, hypermethylation of p16/Ink4a and p19/Arf followed by gene-silencing and loss of p16 and p19 proteins in mesothelioma are noted after intra-peritoneal injection to mesothelioma-inducing CNT and asbestos Chernova et al. [15]. Considering above-mentioned background, epigenetic alterations are important players in the CNT-toxicity and disease outcome.

We previously reported DNA methylation alterations at the gene-specific level in human monocyte cells after 24 h incubation with CNTs (at the concentrations of 25 and 100 μg/ml) [16]. However, CNT-type-specific DNA methylation alterations could not be determined in the monocytes, which play a role in immune response. Bronchial epithelial cells are the first barrier when CNTs reach the lungs after inhalation at occupational settings. Since CNTs are likely to deposit on the lung tissue, studying DNA methylation and subsequent gene expression alterations in these cells will elucidate possible forthcoming adverse effects upon exposure.

The mechanism of CNT-induced epigenetic alterations are currently not known. Epigenetic alterations may be caused by direct CNT-nuclear interactions which might affect the DNA methylation changes. We hypothesized that differential nuclear deposition will affect the epigenome. The following points were assessed considering similarities and differences of MWCNTs and SWCNTs: 1) CNTs localisation in nuclei of 16HBE cells. 2) Identification of global and genome-wide, gene-specific DNA methylation and expression alterations. 3) Identification of aberrant methylation and expression on functionally important genes and cellular networks in order to predict the adverse effects of CNTs.

## Results

### Characterisation of Cnts

**Fig. 1:**
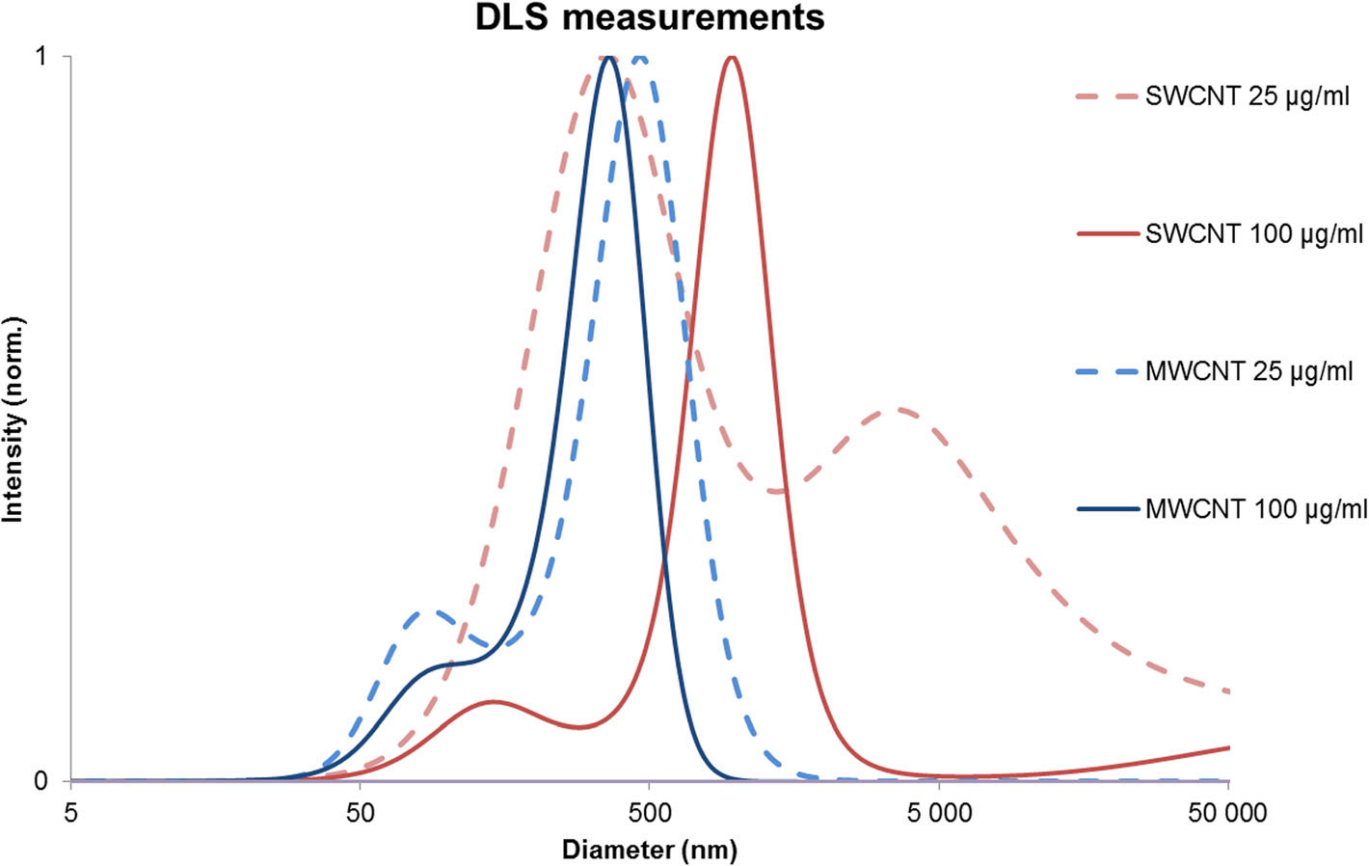
DLS measurements of MWCNTs and SWCNTs

MWCNTs and SWCNTs were obtained from the European Commission Joint Research Centre (JRC, Ispra, Italy) and the National Institute of Technology (NIST, Gaithersburg, Maryland, USA), respectively. The nanomaterials were characterized in detail by the manufacturers [17, 18] and previously summarized in our published study [16]. All characteristics of CNTs are summarized in Additional file 1: Table S1. In brief, the diameters of MWCNTs are approximately 11 nm and the length is on average 846 nm. The diameters of SWCNTs are about 0*.*8 nm and the pristine length is 8000 nm. The CNTs have a high purity (> 95% carbon) but rare elemental impurities are detected. The dynamic light scattering (DLS) analyses (Fig. 1) revealed that CNT dispersions remained stable at the experimental concentrations and that the average size was approximately 50 nm to 500 nm. The size distribution at high dose SWCNTs (100 μg/ml), showed a large peak between 500 nm to 5000 nm and a minor peak between 50 nm to 500 nm. These results might indicate increased agglomeration of the SWCNTs. We did not detect endotoxin contamination in the MWCNTs and SWCNTs dispersions, measured by an established protocol [19].

### Nuclear localisation of CNTs

First of all, we investigated whether fibres can reach the nuclei of the cells. Any physical interaction between CNTs and nuclei might define their epigenetic effects. Label-free detection of CNTs was carried out by imaging their white-light generation under femtosecond pulsed laser illumination as previously described by Bové et al. for carbon black particles [20]. To validate the technique for imaging CNTs, a calibration curve of spiked ultrapure water containing known CNTs concentrations (25 and 100 μg/ml) was made and measured under identical imaging conditions (see supplementary information for a detailed description). A linear relation was observed (*R*^2^ = 0*.*90) between the amount of added and detected CNTs (Additional file 1: Figure S1). This indicated that the described technique is also applicable for detecting CNTs in solution.

**Fig. 2:**
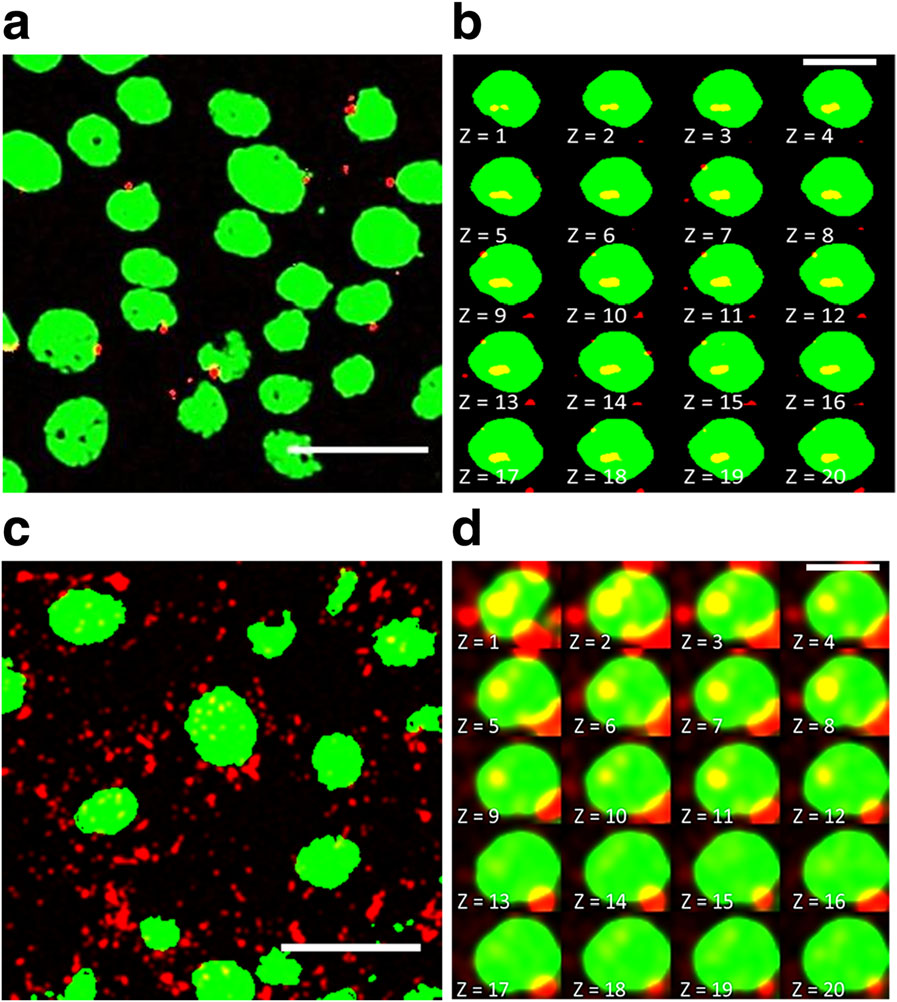
Nuclear deposition of MWCNTs (**a** and **b**) and SWCNTs (**c** and **d**). Maximum projections (**a** and **c**) and views of z-stacks from top to bottom of nucleus (**b** and **d**) CNTs are shown in red but when co-localized with the nucleus they appear yellow and nucleus of a 16HBE cell is shown in green. Scale bars in A and C = 40 μm, scale bars in each panel of the composition of B and D = 15 μm

**Fig. 3:**
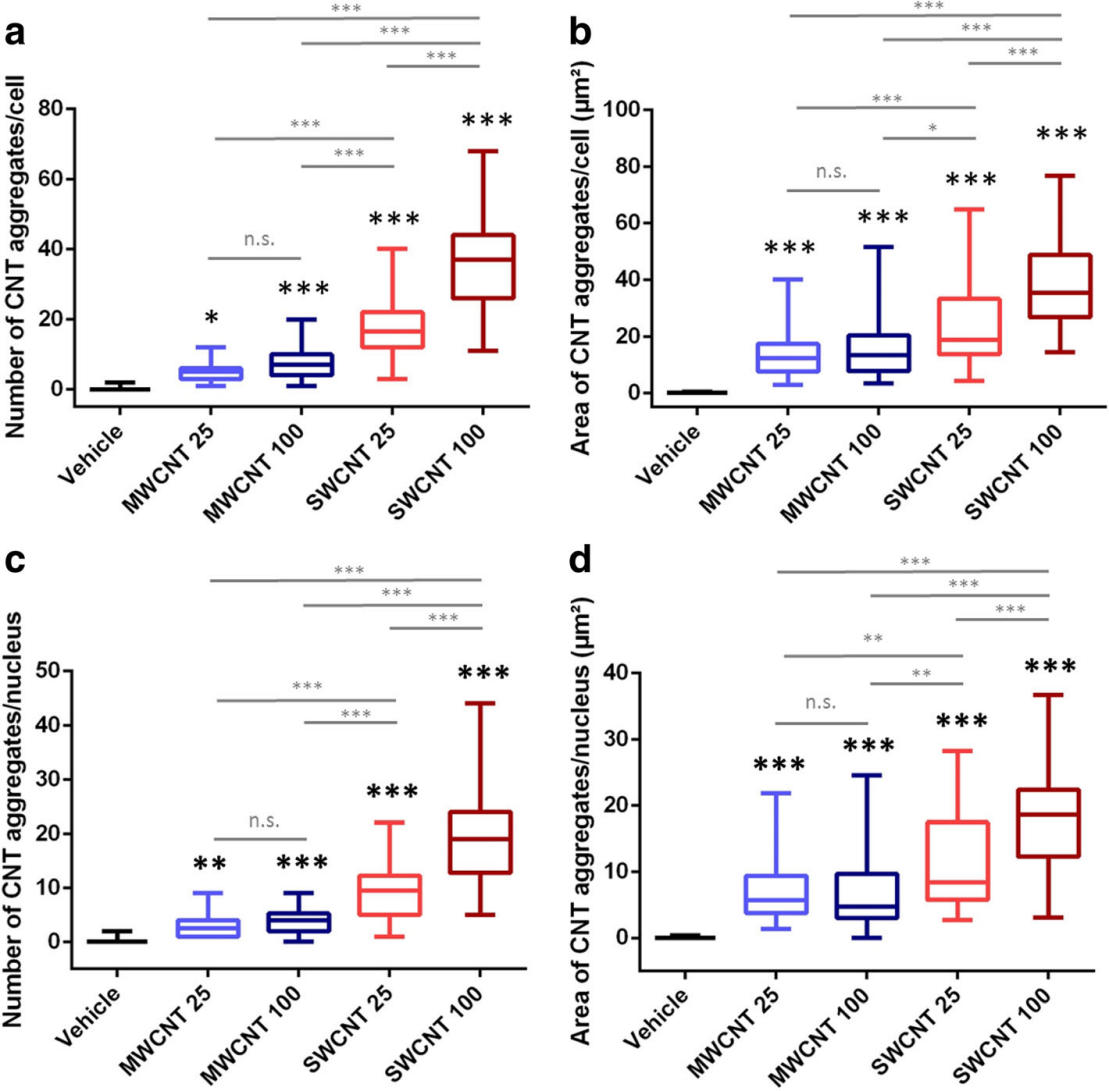
Quantitative analysis of the cellular and nuclear deposition of MWCNTs and SWCNTs. **a** Number of CNT aggregates per cell; **b** total area of CNT aggregates per cell (μm^2^); **c** number of CNT aggregates per nucleus, **d** total area of CNT aggregates per nucleus (μm^2^). All conditions were significantly different from the control condition (vehicle) and from each other (one way ANOVA, Tukey multiple comparison) except for the different doses of MWCNTs [not significant (n.s.)]. The box plots represent median and quartiles, and the whiskers represent the 1.5 interquartile range of the lower and upper quartile (*N* = 50 for each condition). 25 and 100 represents exposure concentrations as μg/ml

Next, the technique was applied to determine the number and total area of CNTs deposited inside both the cells and their corresponding nuclei. In Fig. 2, representative images are shown of both types of CNTs partially attached to or inside of the nuclei of the cells (for MWCNTs Fig. 2a-b and for SWCNTs Fig. 2c-d, Additional files 2-5: Video S1- S4). CNTs in the nucleus is demonstrated by selected z-stack slice images throughout the nucleus from top to bottom (Fig. 2b for MWCNTs, Fig. 2d for SWCNTs) and representative video of these images (Additional file 2: Video S1 and Additional file 3: Video S2). Quantitative analysis for the measurement of CNT number and total area (total area of aggregates/ cell or nucleus in μm^2^) was performed using 2D images. Quantitative analysis (Fig. 3) revealed that: i) Both CNTs were internalized in the cell and in the nucleus in a dose-dependent way (Fig. 3a-d); ii) There is a significant higher cellular uptake (by means of number and total area of aggregates) for SWCNTs compared to MWCNTs (Fig. 3a-b). iii) There is a significant higher nuclear uptake (by means of number a total area of aggregates) for SWCNTs compared to MWCNTs (Fig. 3c-d). However, the ratio nucleus/cellular uptake (data not shown) is similar for the two types of CNTs.

Representative videos, which show single nuclei with deposited CNTs through z-stack images taken from top to the bottom and turning around their y-axes, also clearly demonstrate the particle type dependent difference in nuclear uptake (see Additional file 2: Video S1 and Additional file 3: Video S2, Additional file 4: Video S3 and Additional file 5: Video S4). In summary, both CNTs were in physical contact with the DNA but SWCNTs were spread in greater amounts over both the nucleus and the cell.

### The cytotoxicity and genotoxicity of CNTs

We studied the cytotoxicity and genotoxicity of CNTs in order to understand possible toxic mechanisms that contribute to epigenetic effects [7, 21]. MWCNTs were found to be non-cytotoxic up to a concentration of 256 μg/ml as assessed by both water-soluble tetrazolium salt-1 (WST)-1 and lactate dehydrogenase (LDH) assays (Additional file 1: Figure S2). SWCNTs induced dose-dependent cytotoxicity at doses higher than 64 μg/ml measured by WST-1 assay, despite, no cytotoxicity was observed by LDH assay. Next, induction of DNA strand breaks by CNTs was investigated (after 3 and 24 h exposure). As demonstrated in Additional file 1: Figure S3, no increase in DNA damage was noted after 3 h of exposure. Interestingly, a non-significant increase in DNA damage after 24 h of SWCNTs was observed (mean difference is 0*.*05 to 0*.*1% for MWCNTs and − 9*.*6 to − 9*.*7% for SWCNTs). As shown in Additional file 1: Figure S4, no dose-dependent increase in micronuclei formation was noted after 24 h of MWCNTs and SWCNTs exposure.

### DNA methylation alterations induced by CNTs

**Fig. 4:**
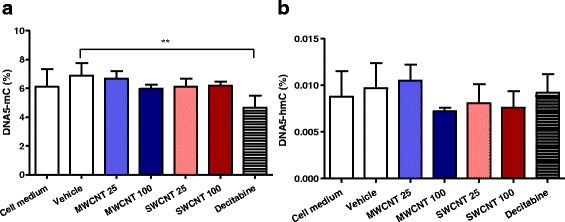
Global DNA methylation and hydroxymethylation analysis after exposure to MWCNTs and SWCNTs. After exposure to MWCNTs and SWCNTs at the selected doses, the whole genomic DNA within the cells were measured by means of (**a**) DNA methylation (5-mC) and (**b**) hydroxymethylation (5-hmC) using LC-MS/MS technique. No significantly different effects were detected between the various conditions. 25 and 100 represents exposure concentrations as μg/ml. Statistics were performed using one-way ANOVA, Dunnett’s multiple comparison test. Only decitabine-exposed cells decreased DNA 5-mC % in comparison to vehicle-exposed cells. ** indicates *p* value < 0.01

The global DNA 5-methylcytosine (5-mC) and 5-hydroxymethylcytosine (5-hmC) alterations at all cytosine residues were analysed using liquid chromatography-mass spectrometry (LC-MS/MS) method, as reported by Godderis et al. [22]. As demonstrated in Fig. 4, exposure to CNTs did not change the global level of 5-mC and 5-hmC in the DNA compared to untreated cells (only cell culture medium and vehicle-treated) after 24 h of exposure. Treatment with 5-aza-2′-deoxycytidine (or known as Decitabine), a hypomethylating agent, induced decreased 5-mdC levels but no overall changes in 5-hmdC.

Although CNTs did not induce alterations at the global level, gene-specific DNA methylation alterations occurred. Whole-genome methylation of CpG sites of the DNA samples (from the cells exposed to CNTs for 24 h) were assessed with the Infinium HumanMethylation450K BeadChip array. The methylation level of the genes was assessed by two different approaches: one based on individual CpG sites across the genomic regions and another based on methylation of the promoter regions. We performed the latter analysis because subtle methylation differences in neighbouring CpG sites, such as those in gene promoters, can also be functional and affect gene expression. Subsequently, next generation RNA-sequencing microarray was performed in order to validate whether certain methylation alterations resulted in differential expression.

### The hierarchical cluster analysis

**Fig. 5:**
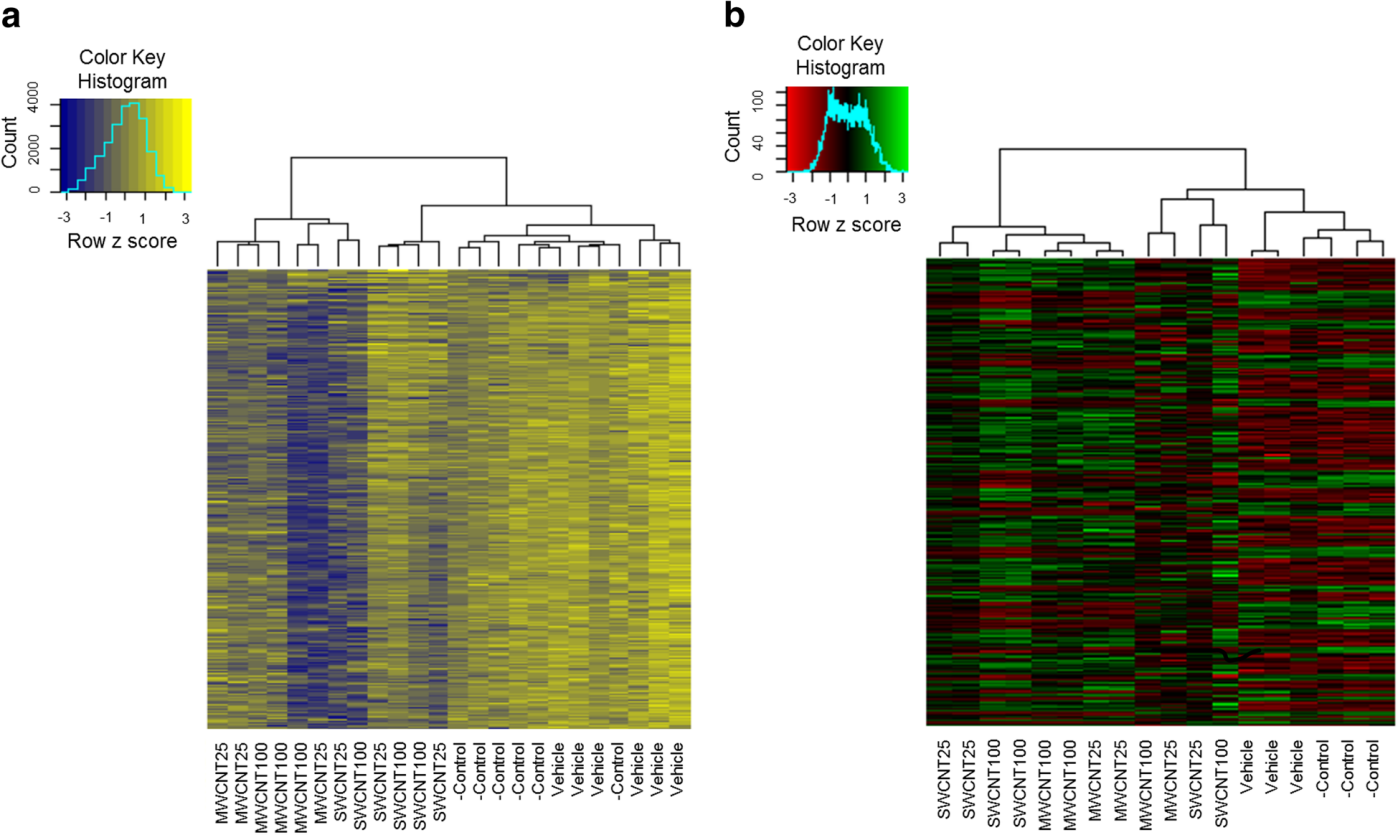
Hierarchical cluster analysis and heat-map to detect differential methylation and expression. The heatmap demonstrates (**a**) differential methylation level ranging between blue and yellow hypomethylation to hypermethylation, respectively) and (**b**) differential expression level ranging between green and red (upregulation to downregulation, respectively) of a substantial number of genes in the MWCNT-treated, SWCNT-treated and untreated [control (only cell culture medium), vehicle (cell culture medium with dispersion medium)] cells. Clustering (based on z scores) of the samples are depicted using the dendrograms at the top of the heatmaps. Different samples names are indicated at the bottom of the heatmaps. Histograms at the top left represent the colour scale reflecting methylation differences and expression differences. 25 and 100 represents exposure concentrations as μg/ml

Hierarchical cluster analysis was performed on differential methylation and expression profiles of untreated and MWCNT- and SWCNT-exposed samples, using the top 500 most differentially methylated and expressed genes, ranked by their FDR-corrected *p* value that referred as *q* value. As shown in Fig. 5a-b, MWCNT- and SWCNT-exposed cells cluster together in a distinct cluster from untreated samples. Although, some irregularities could be seen among the same exposure groups, this is possibly due to batch effect which was statistically corrected for downstream analyses. As explained in supplementary information (see supplementary information, dose specific analysis of the epigenetic data), dose-dependent difference within each CNT remained vague.

### Differentially methylated and expressed genes

After exposure to MWCNTs, 3340 differentially hypomethylated promoter regions were identified, corresponding to 2398 genes (since some genes are characterized by alternative splice variants with same gene promoters). No differential methylation alterations at individual CpG sites were detected. Exposure to SWCNTs resulted in hypomethylated gene promoter regions located at five different genes: A-kinase anchoring protein 8 like (*AKAP8L*), forkhead box K2 (*FOXK2*), eukaryotic translation initiation factor 4E (*EIF4E*), small nucleolar RNA U13 (*snoU13*) and *RP11-223 l10.1*. When assessing individual CpG sites, 589 differentially methylated single CpG sites were identified of which 493 were hypomethylated and 96 hypermethylated. These CpG sites were located on 501 different genes.

Exposure to MWCNTs induced 4028 differentially expressed genes, with 2446 of them upregulated (*log*_2_*FC* > 0) and 1582 of them downregulated (*log*_2_*FC* < 0). Exposure to SWCNTs induced 4964 differentially expressed genes with 2751 of them being upregulated and 2213 of them being downregulated.

### Correlation between gene methylation and expression

**Fig. 6:**
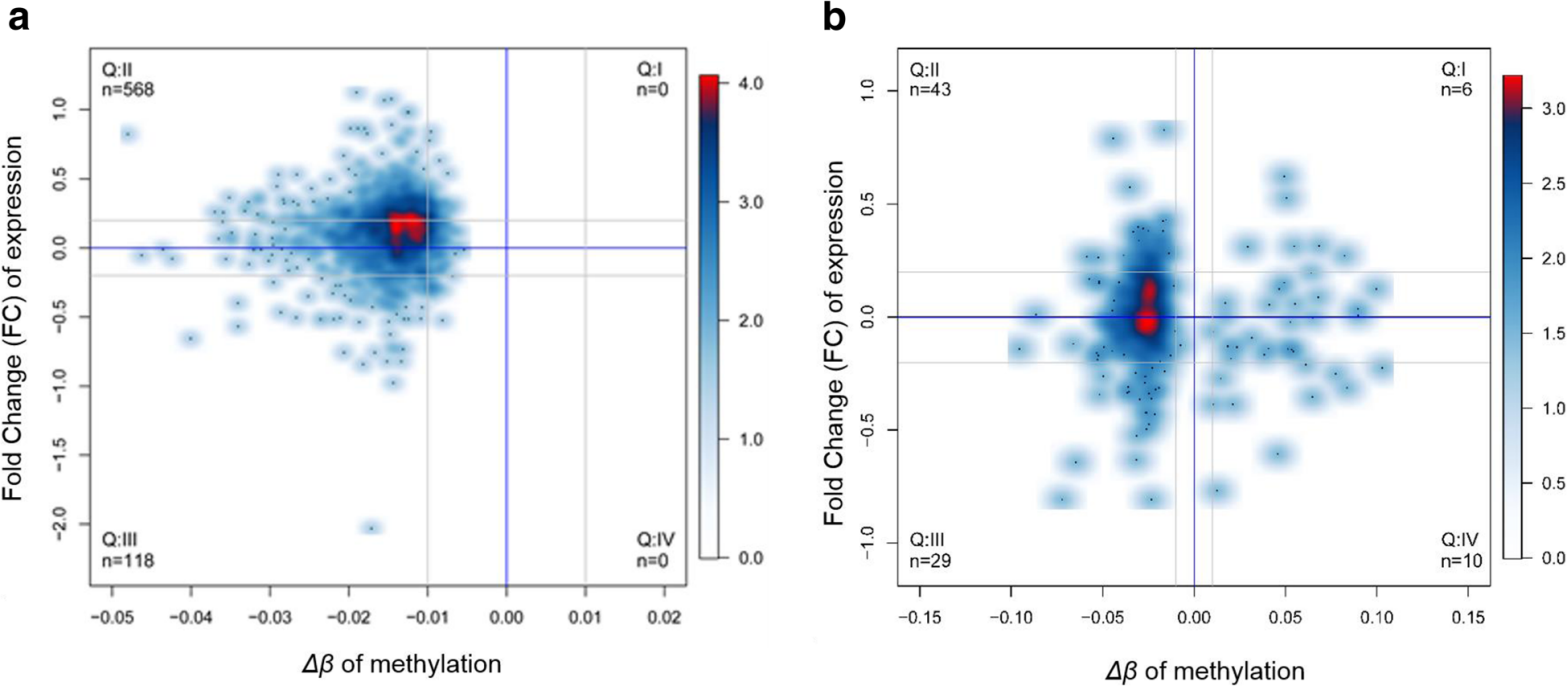
Integrated analysis of gene expression and DNA methylation changes in CNTexposed 16HBE cells. Analysis of gene expressions with significant differential methylation: (**a**) using genes with differentially methylated gene promoters after exposure to MWCNTs and (**b**) using genes with differentially methylated CpG sites after exposure to SWCNTs. *log*_2_*FC i*n mean gene expression are plotted against mean *Δβ* values of individual CpGs and gene promoter regions between CNT-exposed and control samples. Densities of values are plotted by using a color scale from light blue (low density) to red (high density). Gray lines indicate relative hypomethylation and hypermethylation (Δβ > 0.01 or *Δβ* < − 0.01) or relative increase and decrease (*log*_2_*FC* > 0.2 or *log*_2_*FC* < 0.2) in gene expression. In quadrant Q I, genes are relatively hypermethylated after CNT-exposure and show increased gene expression [(A) *N* = 0, (B) *N*= 6]; Q II, genes are relatively hypomethylated after CNT-exposure and show increased gene expression [(A) *N*= 568, (B) *N* = 43]; Q III, genes are relatively hypomethylated after CNT-exposure and show decreased gene expression [(A) *N*= 118, (B) *N*= 29]; Q IV, genes are relatively hypermethylated after CNT-exposure and show decreased gene expression [(A) *N* = 0, (B) *N* = 10]

To ascertain whether differential methylation changes of individual CpG sites or gene promoters were aligned with gene expression changes after exposure to MWCNTs and SWCNTs, scatter plots were generated. In Fig. 6a-b the association between methylation and gene expression alterations are depicted using mean *Δβ* values and mean *log*_2_*FC* for all differentially methylated genes. Only significant differentially methylated gene promoters [FDR corrected *p* value (q value) < 0.05] were mapped. The *Δβ* value of gene promoters in MWCNT-exposed cells ranged between − 0.05 to 0 (indicating only hypomethylation) whereas in SWCNT-exposed cells, they ranged between − 0.10 to 0.10 (indicating hypomethylation and hypermethylation). Although the differential methylation was comparably weaker in comparison to SWCNT-exposed cells, hypomethylation at the gene promoters (*Δβ* < − 0.01) was strongly associated with increased gene expression (*log*_2_*FC* > 0.2) for MWCNTs-exposed cells using another technique: RNA sequencing. Smaller associations between single CpG site methylation and gene expression changes were noted for SWCNTs-exposed cells. Since only five differentially methylated gene promoter regions were identified after SWCNT exposure no profound analysis could be performed.

### Gene-function analyses

1. MWCNTs: Using differentially methylated genes after exposure to MWCNT (total 2398 genes), functional – Gene Ontology (GO) analysis is reported in Additional file 1: Table S2 and Gene functional classification is reported in Additional file 1: Table S3. GO enrichments were involved in multicellular organism development and regulation of small Guanosine-5′-triphosphate (GTP)ase mediated signal transduction pathways whereas no enrichment using Kyoto Encyclopedia of Genes and Genomes (KEGG) pathway database was noted. Functional gene classification analyses resulted in following gene groups (top 5 clusters): pleckstrin homology-like domain, GTPase activator activity, positive regulation of GTPase activity/metal binding/zinc finger, nucleus/DNA binding and protein binding groups, in consistency with the GO analysis.

Using differentially expressed genes after exposure to MWCNT (in total 4028 genes), functional – GO analysis is reported in Additional file 1: Table S4, KEGG pathway analysis is reported in Additional file 1: Table S5 and gene functional classification analysis is reported in Additional file 1: Table S6. Enriched GO terms were noted in protein transport and phosphorylation ontologies, which play a crucial role in nearly all cellular signalling, enabling activation or deactivation of the proteins. Enriched KEGG pathways were noted in metabolic pathways, Human t-lymphotropic virus-1 (HTLV-I) infection and with close proximity of significance tumour protein 53 (TP53) signalling pathway. Functional gene classification analyses resulted in the following top five clusters: phosphoproteins/transcription, transcriptional regulation DNA-templated, zinc finger/metal binding, chromosome/phosphoproteins, and WD repeat domain groups.

2. SWCNTs. Using differentially methylated genes after exposure to SWCNTs, functional –GO analysis is reported in Additional file 1: Table S7, KEGG pathway analysis is reported in Additional file 1: Table S8 and gene functional classification analysis is reported in Additional file 1: Table S9. The GO analysis resulted in enrichment of multiple glucuronidation processes. The KEGG analysis resulted in enrichment of wide-range of metabolic pathways. Functional gene classification analyses resulted in following gene groups (4 clusters): Uridine 5′-diphospho-glucuronosyltransferase (UDP glucuronosyltransferases), transmembrane proteins, zinc-finger C2H2 like, immunoglobulin subtype/signal peptides.

Using differentially expressed genes after exposure to SWCNTs (in total 4964 genes), functional – GO analysis is reported in Additional file 1: Table S10, KEGG pathway analysis is reported in Additional file 1: Table S11 and gene functional classification analysis is reported in Additional file 1: Table S12. Enriched GO terms were noted in protein transport, DNA damage response, cell cycle and cell migration and adhesion ontologies. KEGG analysis metabolic, endocytosis, cell cycle and p53 signalling.

Functional gene classification analyses resulted in following top five clusters: nucleic acid binding, sister chromatid cohesion, ankyrin repeats, transcriptional regulation, ATP-binding groups.

**Fig. 7:**
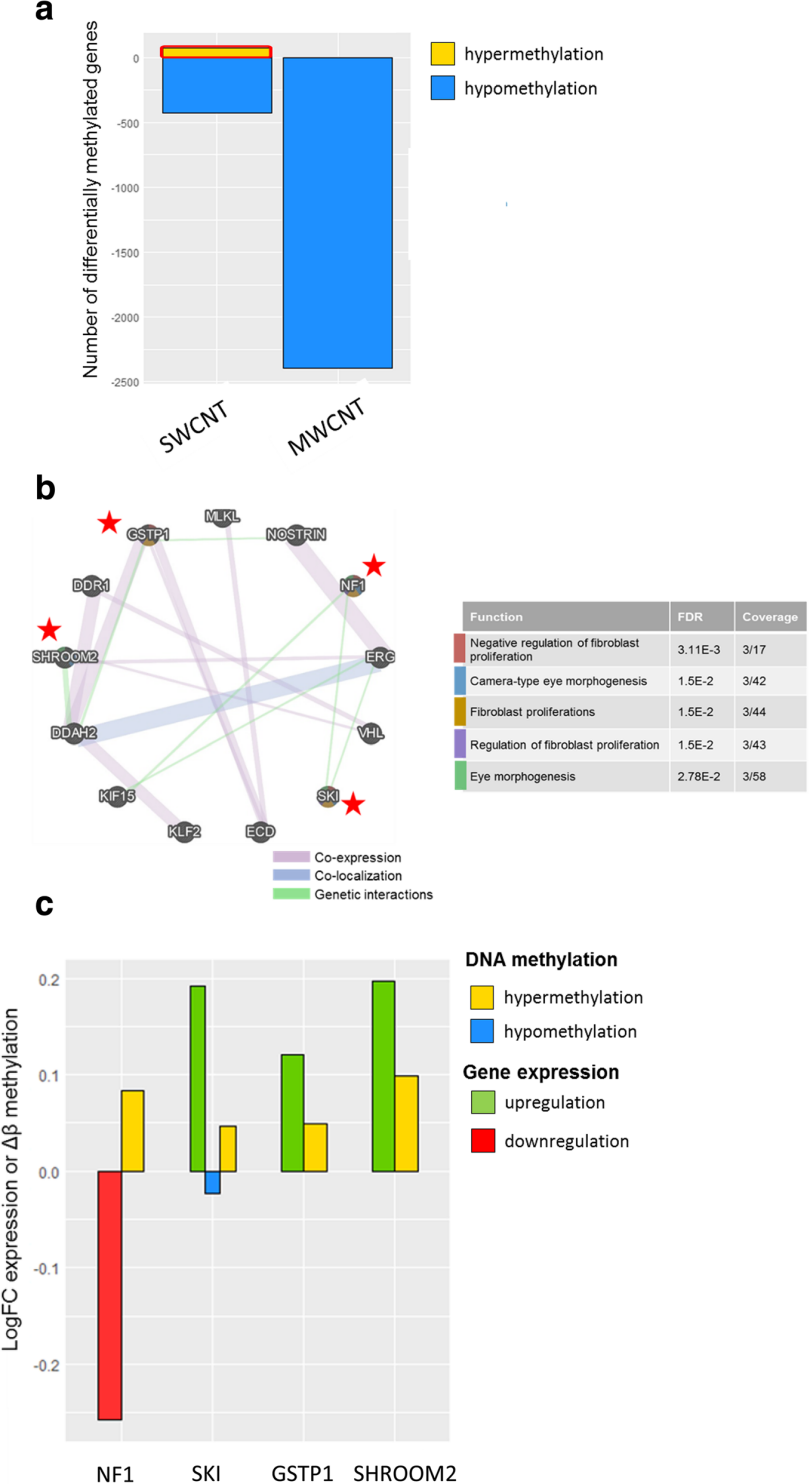
Differentially hypermethylated genes by their CpG site after exposure to SWCNTs. **a** set of differentially hypermethylated genes (by CpG sites) were selected. **b** Selected set of genes were proceeded for functional analysis using web-based GeneMania tool (http://www.genemania.org). The genes, which involve in the enriched networks are indicated with a red star and were further investigated for gene expression status (**c**)

Since CpG specific hypermethylation was only noted by SWCNTs and hypermethylation was generally linked with gene silencing in tumorigenesis, we further investigated this set of differentially hypermethylated genes [23, 24]. As demonstrated in Fig. 7, genes in developmental and cellular proliferation pathways were identified as follows: SKI proto oncogene (*SKI, gene body and 3’UTR*), neurofibromin 1 (*NF, gene body*), shroom family member 2 (*SHROOM2, 5’UTR*), glutathione s-transferase pi 1 (*GSTP1, 1st exon and 5’UTR*), kruppel like factor 2 (*KLF2, gene body*), von hippel-lindau tumor suppressor (*VHL, TSS200*), discoidin domain receptor tyrosine kinase 1 (DDR1, TSS1500 and 5’UTR), mixed lineage kinase domain like (*MLKL, TSS1500*), dimethylarginine dimethylaminohydrolase 2 (*DDAH2, TSS1500*), nitric oxide synthase trafficking (*NOSTRIN, 5’UTR and 1st exon*), ERG ETS transcription factor (*ERG, gene body*), ecdysoneless cell cycle regulator (*ECD, TSS200*), kinesin family member 15 (*KIF15, TSS200*). Using the network analysis, regulation of fibroblast proliferation by alterations on *GSTP1, SKI, NF1* genes and eye morphogenesis by alterations on *NF1, SKI* and *SHROOM2* genes were identified. By checking expression values of these three differentially methylated genes only NF1 showed hypermethylation and downregulation. Of note, aberrations on NF1 gene have been noted in lung adenocarcinoma and *NF1* mutations are observed with TP53 alterations [25–27].

### RT-PCR analysis of the selected genes

**Fig. 8:**
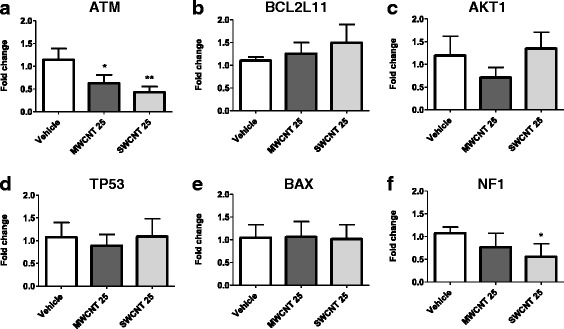
RT-PCR analysis of the selected genes, **a**) ATM, **b**) BCL2L11, **c**) AKT1, **d**) TP53, **e**) BAX, **f**) NF1, after low dose (25 μg/ml) exposure of MWCNTs and SWCNTs. * indicates *p* < 0.05, using t-test, two-tailed (95%confidence)

Following our analyses, we used RT-PCR to validate our data analysis according to the pathways that are proposed by our data mining analysis. We focused on lowest dose of exposure (25 μg/ml) in order to identify early contributor of potentially altered pathways such as p53 signalling, DNA damage response and cell cycle, by investigating the alterations of the hub-genes (i.e. ATM, P53 and AKT1) and downstream products (i.e. NF1, BCL2L11 and BAX). Demonstrated in Fig. 8, we noted downregulation of the expression of ATM gene for both CNTs, upregulation of the expression of BCL2L11 for SWCNTs and downregulation of the expression of NF1 for SWCNT genes, as expected. Consequently, it can be concluded that ATM, BCL2L11 and NF1 genes which are involved in up- and downstream pathways of tp53, DNA damage response, apoptosis and cell cycle signalling are differentially methylated and expressed after CNT exposure and may potentially contribute alteration of proposed signalling pathways.

## Discussion

In this study, we observed greater aggregation and particle deposition of the cell nucleus after exposure to SWCNTs. Comparably, MWCNTs were relatively smaller in size and localized over the nucleus less than SWCNTs. In addition, increasing concentrations of MWCNTs did not lead to significantly different values in the area and aggregates of the MWCNTs near the nucleus. As a matter of fact, SWCNTs are more in contact with the cell nucleus compared to MWCNTs regardless of the increasing concentrations. Although, such nuclear-CNT interactions will have an on various aspects of nucleus, here, we first aimed to identify, how nuclear deposition will have an impact on the genetic and epigenetic toxicity of the CNTs.

In the literature, more DNA methylation alterations on CpG sites were noted after four weeks of exposure in comparison to two weeks of exposure Sierra et al., [14]. This observation of low number of DNA methylation changes after short period of exposure is in correlation with our study, since, we detected vague response on dose-specific analysis. Therefore, we improved the power of the statistics by combining two different doses, however more replicates may be necessary in future studies. In addition, Sierra et al. observed that most of the hypomethylated genes after two weeks exposure became hypermethylated after four weeks of exposure Sierra et al., [14]. This may indicate that possible DNA demethylation and gene-silencing mechanisms occurred during the prolonged exposure. Of note, in cancer cells, global loss of DNA methylation and increased gene-specific methylation have been noted (Baylin and Ohm, [28]; Esteller, [29]). Gene-silencing through increased methylation increase the risk of spontaneous mutation (Li and Zhang, [9]). Regarding the loss of DNA methylation, currently two main processes are known: active and passive DNA demethylation processes in the development (Li and Zhang, [9]). Active DNA demethylation refers to removal of methylation from the 5-mC by enzymatic processes. Alternatively, passive DNA demethylation refers to alterations or inhibition of DNA maintenance genes, such as DNMT1, leading the loss of DNA methylation on cytosine residues. DNA demethylation may also occur via TET-mediated 5-mC oxidation leading to formation of 5-hmC. However, in this study, we did not detect any alterations on DNMT1 and TET genes (based on our RNA sequencing data) nor in global 5-hmC methylation levels. Other mechanisms which may induce changes in DNA methylation machinery may require further investigation such as histone modifications, miRNA mechanisms, genotoxicity, oxidative stress or inflammation. For instance, early stress response on key genes such as ATM may initiate epigenetic machinery through alterations on DNA damage response. Of note, we observed downregulation of ATM gene at the lowest dose of exposure, which may result in impaired cell cycle and DNA damage response mechanism in the cell, resulting in increased genotoxicity, leading to changes in methylation. Importantly, here, we show CNTs localized in the cellular nucleus, which could lead to loss/gain of DNA methylation by mechanical interference.

Considering genotoxic endpoint, we observed more pronounced cyto- and genotoxicity by SWCNTs compared to MWCNTs. The same type of SWCNTs have been already associated with DNA damage and alterations on cellular signalling pathways Pacurari et al., [30]. In agreement with our findings, non-cytotoxic and non-genotoxic effects of this type of MWCNTs have been reported in an OECD project Hannu Norppa [31].

From literature, we know that DNA damage and epigenetic factors are in association. For instance, it is noted that knock down of the DNA methyl transferase 1 (*DNMT1*) gene, a major regulator of DNA methylation maintenance, activates wide-range of genotoxic check point genes [32, 33]. DNA hypomethylation on genes such as DNMT1 and stress response might trigger activation of DNA repair pathways. On the other hand, gene silencing on tumour suppressor genes may lead to toxic responses in the cell. The specific interaction between genotoxicity and epigenetic alterations require further mechanistic research.

From the DNA methylation/gene expression point of view, the differences in DNA methylation after exposure to MWCNTs and SWCNTs can be summarised via three main points.

*First*, DNA methylation alterations on gene promoter regions were observed after exposure to MWCNTs, while methylation changes at the single CpG sites (and some gene promoters) were observed after exposure to SWCNTs. Methylation alterations on gene promoter regions can be a result of indirect interactions of MWCNTs with the cytosolic proteins and RNAs that in turn affect the epigenetic machinery within the DNA level. Our analysis shows that SWCNTs have significantly more physical contact with nuclei compared to MWCNTs. Since SWCNTs are likely to be endocytosed into cellular vesicles, they may not be in contact with the cytosolic proteins or RNAs but rather directly in touch with the nucleus. This might be a valid explanation for the high number of CpG site methylation (alterations) after SWCNTs exposure and gene promoter hypomethylation after exposure to MWCNTs.

As expected, methylation changes of gene promoters have a different (stronger) effect on the expression profile compared to the methylation of the CpG sites. This is due to the fact that hypomethylation on gene promoter regions will foster the binding of transcription factor and activate the gene transcription.

*Second*, only hypomethylation was seen after exposure to MWCNTs whereas both hypomethylation and hypermethylation (to a smaller extent than hypomethylation) was seen after exposure to SWCNTs. Hypomethylation may lead to the activation of a cellular response that may trigger damage repair within the cell. Therefore, hypomethylation might serve as a buffer response of the cell toward toxic exposures such as nanoparticles. It might also be related with the increased inflammation that may trigger an activation of the cellular response. On the other hand, hypermethylation is associated with the gene silencing. The gene silencing has been typically observed in diseased tissues such as tumours [34]. We observed that a functionally important gene such as NF1 (a gene which serves as a tumour suppressor and acts in fibroblast proliferation) was both hypermethylated and downregulated. However, it is important to note that the study addresses the in vitro conditions when the exposure is limited to 24 h. The nuclear deposition of CNTs may alter in prolonged exposure or in in vivo conditions where the active clearance of the particles are present.

*Third*, both CNTs altered similar pathways by means of gene expression and methylation alterations. When only methylation alterations were concerned, overrepresentation at GTPase mediated signal transduction, multicellular organism development and inflammatory pathways were seen after exposure to MWCNTs, whereas overrepresentation on glucuronidation, metabolic and endocytosis processes were seen by SWCNTs exposure.

It has been shown that differences in physicochemical structure of CNTs affect their toxic properties and potential disease outcome. For instance, long, needle-like MWCNTs are shown to induce mesothelioma in rodents, like asbestos fibres, through asbestos-like toxicity mechanisms [15, 35]. This was not observed by their shorter counterparts. Therefore, it is crucial to note that differences observed by different types of CNTs might well be related to their size and shape. For instance, SWCNTs are long fibres with an extremely small diameter. Although, they do not have a needle-like shape, they keep their fibre structure and high surface area, which makes them more reactive. In contrast, MWCNTs that have been used in this study, are relatively short with an average diameter of 11 nm, allow them to be easily internalized and cleared out in/from the cells. This explains the greater amount of SWCNTs localised in the nucleus and the hypermethylation observed by SWCNTs only.

**Fig. 9:**
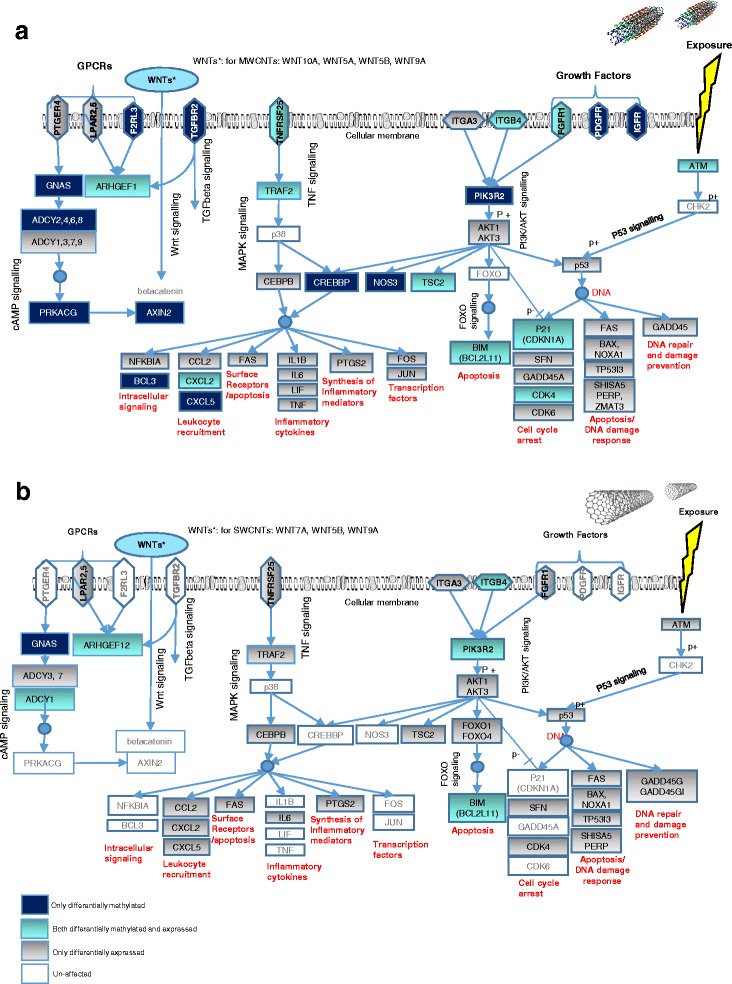
An overview of functionally altered pathways. The diagram demonstrates the functionally altered cell signalling pathways after exposure to **a** MWCNTs and **b** SWCNTs. The grey coloured boxes which consist the differentially expressed genes, the dark blue colour indicates only differentially methylated genes, turquoise blue colour indicates both differentially methylated and expressed genes within the mentioned pathways. Genes that are written in white boxes are unaffected. Blue circles indicate DNA. ‘p+’ and ‘p-’ indicate phosphorylation and de-phosphorylation, respectively

Nevertheless, common pathway alterations in MWCNT- and SWCNT-treated cells were noted and summarised. In Fig. 9, an overview of the most altered cell survival pathways such as p53, DNA damage response, cell cycle, phosphatidylinositide 3 kinases-protein kinase B (PI3K-AKT) pathway, mitogen-activated protein kinase (MAPK) signalling, by differentially methylated and/or expressed genes were visualized. This analysis are performed in order to give a better visualization of functionally altered pathways after exposure to MWCNTs and SWCNTs.

P53 is a critical tumour suppressor gene that orchestrates cellular stress response such as cell cycle, DNA repair, and apoptosis. The classical alteration of p53 signalling includes alterations of ATM gene, which phosphorylates checkpoint kinase 2 (CHK2) and phosphorylation of p53 from several sites, after exposure to DNA damaging agents. Activated (phosphorylated) P53 stimulates downstream cellular responses such as cell cycle. The cell cycle signalling involves cyclin dependent kinase inhibitor 1A [CDKN1A (p21)] and cyclin dependent kinases (CDKs) such as cyclin dependent kinase 4 (CDK4) and cyclin dependent kinase 6 (CDK6) and DNA damage repair. The cell cycle signalling is crucial for the cell to maintain normal rate of proliferation. Therefore, the cell cycle deregulation in the cell increases the risk of cancer initiation by altering the proliferation rate. The studies on the relevance of cell cycle in cancer point out deregulation of p21 and CDKs (CDK4 and CDK6) [36, 37]. The potential role of p53 in malignant transformation of lung epithelial cells after continuous SWCNT exposure and activation of pAkt/p53/Bcl-2 signalling axis was noted by whole genome gene expression analysis [38, 39]. In our analysis we noted alterations on TP53, DNA damage response and cell cycle signalling induced by CNTs. Genes such as fas cell surface death receptor (FAS), BCL2 associated x, apoptosis regulator (BAX), NADPH oxidase activator 1 (NOXA1), tumour protein P53 inducible protein 3 (TP53I3), shisa family member 5 (SHISA5), PERP TP53 apoptosis effector (PERP), TP53 were differentially expressed by both type of CNT-exposure. Differential methylation and expression on ATM, p21 and CDK4 genes were noted in the cells exposed to MWCNTs. For SWCNTs, only differential gene expression of ATM and CDK4 genes were noted.

In addition, PI3K-AKT signalling can contribute to the alterations in p53 signalling. PI3K group of genes including phosphoinositide-3-kinase regulatory subunit 2 (PIK3R2) gene, were activated by receptor tyrosine kinases (RTKs) such as fibroblast growth factor receptor 1 (FGFR1), platelet-derived growth factor receptor beta (PDGFR), insulin-like growth factor 1 Receptor (IGFR). PI3K phosphorylates AKT genes, which stands upstream or various cellular processes such as p53 signalling, cellular differentiation and apoptosis. Upon activation of the The forkhead box O (FOXO) transcription factors, BCL2 like 11 (BCL2L11, also known as BIM) acts as a regulator of apoptosis. Exposure to SWCNTs induced differential methylation and expression on PIK3R2 gene and both types of CNTs altered methylation and expression state of BCL2L11 gene. Exposure to MWCNTs induced differential methylation on *IGFR, PDGFR* and methylation/expression changes on FGFR1 genes.

Concerning MWCNTs, increased inflammatory response was evident. For instance, the KEGG pathway ‘HTLV infection’ was noted for MWCNT-exposed cells. In addition, alterations on tumor necrosis factor receptor superfamily member 25 (TNFRSF25) and tumor necrosis factor receptor associated factor 2 (TRAF2), which mediate signal transduction and activate inflammatory response through MAPK and nuclear factor-kappa B (NF-κB) pathways, were noted to be both differentially methylated and expressed. In addition, differential expression of inflammatory cytokines such as interleukin 1-beta (IL1-β), interleukin 6 (IL6), leukemia inhibitory factor (LIF) and tumour necrosis factor (TNF) was induced by MWCNTs whereas only differential expression of IL6 was induced by SWCNTs. Differential expression on nuclear factor-kappa B inhibitor alpha (NFKBIA) and differential methylation on B cell lymphoma 3 (BCL3) gene was induced by MWCNTs. Likewise, differential methylation and expression on chemokine (C-X-C motif) ligand 2 (CXCL2) gene was induced by MWCNTs whereas exposure to SWCNTs caused only differential expression.

These results might also explain epigenetic contribution to in vivo CNT-mediated adverse effects. For instance, exposure to SWCNTs caused fibrogenic pulmonary responses in rodents [40–43]. MWCNTs (NM400) showed adverse pulmonary effects in mice, summarized as increased cytokine formation such as IL1-β, IL6, TNF, Chemokine (C-X-C motif) ligand 1 (CXCL1), C-C Motif Chemokine Ligand 2 (CCL2), C-C Motif Chemokine Ligand 4 (CCL4), and C-C Motif Chemokine Ligand 5 (CCL5) and increase in the genes related to cellular adhesion, inflammation, oxidative stress and DNA damage repair [44, 45].

In this study, we used two different microarrays and generated tremendous data which can be used to elucidate and speculate alterations in important pathways. Variances and irregularities between samples could be seen due to many reasons and these changes might affect the observed results. These reasons include experimental protocols, aging of the cells, and the batch effects that occurs during microarray processing. In order to overcome these issues, the cells were harvested at the same passage number (passage 4) and batch correction was applied during the bioinformatics analyses.

Collectively, we demonstrate localisation of CNTs (SWCNT > MWCNT) in cellular nucleus in a label-free way and CNT induce subtle epigenetic and gene expression alterations in vitro at the acute phase. Although differences between two CNTs were noted, similar pathways altered by two types of CNTs. Our results are consistent with previous studies in which we noted DNA methylation alterations on ATM gene in mouse lungs after intra-tracheal CNT administration and in blood samples of the workers who have exposed to MWCNTs [11, 46]. Downregulation of ATM gene may regulate DNA damage response, DNA damage checkpoints, cell cycle, and p53 signalling and these signalling are enriched in our data analyses. Overall, our data provide possible pathways and gene sets which may alter by CNT exposure at the acute phase. Importantly, there is a need to investigate epigenetic and functional outcomes of the proposed mechanisms after a longer exposure period.

## Conclusions

In the current study we have shown that SWCNTs are engulfed and distributed superiorly compared to MWCNTs, at both the cellular and nuclear level. Accordingly, increased cytotoxicity and genotoxicity for SWCNTs were found. Although, no global DNA methylation nor hydroxymethylation alterations were seen for both types of CNTs, whole-genome DNA methylation alterations were particle type dependent. Transcriptomic profiles of CNTs show differential regulation of a diverse set of genes but alterations in similar pathways (i.e. DNA damage, DNA damage repair, tp53, cell cycle, protein phosphorylation). In particular, exposure to SWCNTs induced fibroblast proliferation and exposure to MWCNTs induced alterations in the genes responsible for inflammation.

It can be postulated that epigenetic mechanisms serve in repair or toxic response to the exposure and might increase the disease susceptibility by hypomethylation or hypermethylation. In particular, specific DNA hypermethylation and gene silencing should be taken in caution hence it might lead to gene silencing at tumour suppressor genes. These differences elucidate cellular epigenetic behaviour of the cells is dependent on type of CNTs and CNT-nuclear interactions. Overall, DNA methylation alterations might result in adverse effects in rodents and human after the inhalation exposure.

## Methods

### Cell cultures and particle preparation

Dulbecco’s Modified Eagle Medium (DMEM), Dulbecco’s Modified Eagle Medium: Nutrient Mixture F12 (DMEM/F12), Hank’s Balanced Salt Solution without CaCl_2_ and MgCl_2_ (HBSS-), phosphate buffered saline without CaCl_2_ and MgCl_2_ (PBS-), penicillin-streptomycin, amphothericin-B, L-glutamine, fetal calf serum (FCS), 0.5% Trypsin-EDTA were purchased from Invitrogen (Merelbeke, Belgium).

16HBE human bronchial epithelial cell lines (16HBE14o-) were provided by Dr. Gruenert (University of California, San Francisco). The cells were cultured in DMEM/F12 supplemented with 5% of FCS and 1% of Penicillin-Streptomycin (10,000 U/ml), L-glutamine (200 mM) and, Amphotericin-B (250 μg/ml), incubated at 37 °C in a 100% humidified atmosphere containing 5% CO_2_. Culture medium was renewed every 2 or 3 days and 2.5 × 10^5^ confluent cells were sub-cultured in a new cell culture T25 flasks. To do this, the cells were enzymatically released by 0.05% trypsin-EDTA solution (diluted in HBSS-).

Stock of cells was generated from the same passage number (passage number 4) and kept in liquid nitrogen to avoid the effect of aging in epi-genotoxicity analysis. Cells from the same stock were grown until passage 4 for epigenetic experiments. When the desired passage number was reached and the cells became confluent, 16HBE14o- cells were seeded in wells of plates at 2 × 10^5^ cells/cm^2^ of density and allowed to attach for 24 h to reach 80% confluence.

CNT suspensions were prepared as described in the European project of Engineered Nanoparticle Risk Assessment (ENPRA) [47]. In brief, CNTs were diluted in Baxter sterile water containing 2% serum to reach a final concentration of 2.56 mg/ml. The suspension was sonicated for 16 min using probe sonication at frequency 22.5 kHz, watt 7.35 W with 50% amplitude (MICROSON XL 2000). Final concentrations were by diluting 1/10 of the intermediate concentrations. Fresh CNT suspensions were prepared before each experiment to avoid aggregation of CNTs. While exposing CNTs, no serum was added in the cell culture medium and each concentration involved 0.02% of serum.

### Physicochemical assessment of CNTs

Two different reference materials of CNT were used, NM400 MWCNTs were obtained from the European Commission Joint Research Centre (JRC, Ispra, Italy) and SRM:2483 SWCNTs were from National Institute of Technology (NIST, Maryland, USA) [17, 18].

Endotoxin determination was performed using ENDOSAFE PTS cartridges (Charles River laboratories, Massachusetts, USA). 1/1000 dispersion from the master solution of CNTs in ENDOSAFE LAL reagent (Charles River laboratories, Massachusetts, USA) water was used.

DLS analysis was performed to gain information about the suspension and aggregation state of the nanomaterials in the cell medium at 25 and 100 μg/ml of concentration.

Transmission Electron Microscopy (TEM) images of the MWCNTs and SWCNTs were previously demonstrated [16].

### Nuclear deposition

Nuclear deposition of MWCNTs and SWCNTs was imaged according to the method that has been reported previously by Bové et al. [20]. The method detects CNTs in a label-free and biocompatible fashion in cellular compartments of interest using femtosecond pulsed laser microscopy. First, before executing cellular experiments, the method was validated for CNTs by measuring spiked ultrapure water with known concentrations of the different nanomaterials using identical imaging conditions as later used for the cellular experiments.

The cells were exposed to MWCNTs and SWCNTs (25 and 100 μg/mL of doses) in 8 well chamber slides for 24 h (Invitrogen, Merelbeke, Belgium) in cell culture medium. Slides were washed five times (to avoid CNTs residuals) with HBSS- and the cells were fixed with 4% paraformaldehyde. Staining was performed by 5 min treatment of SYBERgold (Invitrogen, Merelbeke, Belgium) diluted 1/20000 times in HBSS-. After the cells were washed three times using HBSS-.

Images were acquired using a Zeiss LSM510 META NLO scan head mounted on an inverted laser-scanning microscope (Zeiss Axiovert 200 M; Zeiss, Germany) and a 40×/1.1 water immersion objective. For imaging the stained nuclei, a 30 mW air-cooled Argon ion laser (LASOS Lasertechnik GmbH, Germany) emitting at 488 nm (~ 3 μW maximum radiant power at the sample) was used as excitation source and a band-pass filter 500–530 nm was used for filtering the emission light. A fixed pinhole size of 100 µm was used. CNTs were visualized by femtosecond pulsed laser excitation (~ 4 mW average laser power at the sample, 810 nm, 150 fs, 80 MHz, MaiTai DeepSee, Spectra Physics, USA) and filtering of the emission signal by a 400–410 nm band-pass filter in the non-descanned mode. The pinhole was opened completely. The resulting 512 × 512 images with a pixel size of 0.44 μm were recorded at a pixel dwell time of 3.2 μs. In addition, three-dimensional z-stacks were acquired throughout the cells every 25 - 30 µm to confirm nuclear deposition (~ 225 × 225 × 30 μm image volume). Images were captured using the AIM 4.2 software (Carl Zeiss).

Images were processed with the image-processing program Fiji (ImageJ v1.47, open source software, http://fiji.sc/Fiji). Prior to the analysis, the cell of interest was cropped in a way that non-engulfed CNTs were excluded from the analysis. A threshold was set to the estimated background value and the number and total area of CNT aggregates inside the cell were measured. Next, the corresponding nucleus was cropped and the number and area of deposited CNT aggregates were determined. In total, 50 cells for each exposure conditions including negative controls were analysed.

### Cytotoxicity

The cell viabilities were analyzed by two different assays, namely WST-1, LDH assays. Two independent assays (with three replicates) were performed according to optimized protocols. For both experiments, cells (2 × 10^5^ cells/cm^2^) were exposed to 4, 8, 16, 32, 64, 128 and 256 μg/ml of MWCNTs and SWCNTs for 24 h and untreated cells were included as negative controls.

In brief, following protocol was applied for WST-1 assay. After the exposure in the 96-well plate, cells were rinsed one time with cell culture medium without phenol red (DMEM). WST-1 solution (Sigma Aldrich, Brussels, Belgium) was diluted 1/20 in DMEM medium and the cells were incubated with this solution for 2 h at 37 °C. Subsequently, the supernatant was transferred to another well plate for absorption measurement of the formazan product at 450 nm optical wavelength. Relative viability was calculated in comparison to untreated (cell medium without serum) cells.

In brief, following protocol was applied for LDH assay. After the exposure, the supernatant was transferred to another plate. The cells were lysed (using 200 μl 0.2% Triton X-100 in PBS+) and incubated for 30 min. The measurement was taken with the addition of the reaction mixture (18.32 mg pyruvate, 21.28 mg NADH, 31.76 mg HCO_3_Na dissolved in 40 ml PBS+) was added on the cells. The results were obtained using a spectrometer, 340 nm wavelength every 15 s for 3 min after 5 s of mixing.

Data analysis was calculated according to the absorbance curve and relative viability was calculated according to untreated controls.

### Comet assay

Induction of DNA strand breaks after 3 h and 24 h exposure to CNTs was assessed using alkaline comet assay [48]. The assay was performed using Trevigen Comet assay kits (Gentaur, Kampenhout, Belgium) according to the manufacturer’s protocol. Due to nanomaterial interference and as well as cell-specific conditions, necessary adjustments were performed. In brief, cells were trypsinized and suspended at 1*x*10^5^ cells/ml HBSS-. 5 μl of cells were mixed with freshly prepared low melting point agarose and immobilized on Trevigen comet assay slides (Gentaur, Kampenhout, Belgium). After cooling, the slides were immersed in lysis solution for 30 min. Subsequently, the slides were immersed in alkaline solution for 15 min to unwind the DNA. Finally, the slides were electrophoresed for 30 min. SYBERgold staining (1/10000 diluted in distilled water) was applied after cell fixation with 70% of ethanol.

The results were analyzed using a fluorescence microscope. 50 comets in the cells from each 2 replicates were measured for DNA damage by means of the % DNA Tail metric using the CaspLab program (casplab 1.2.3b2) according to the following formula:

$$ \mathrm{DNA}\ \mathrm{Tail}\ \left(\%\right)=\frac{\mathrm{Tail}}{\mathrm{Head}+\mathrm{Tail}}\times 100 $$

The means of the two medians for each exposure type were represented.

### Micronucleus assay

The micronucleus assay was adapted according to the OECD guidelines [49, 50]. Untreated cells were used as negative control and 0.6 mM mitomycin (Sigma Aldrich, Brussels, Belgium) was used as positive control. Mitomycin increased the number of micronuclei (mean 48.5, sd: 21.8, in 500 cells). No Cytochalasin B (CytB) was used. After exposure, the cells were further incubated with cell culture medium for 48 h (corresponding 1.5 doubling time). The number of micronuclei (MNs) in 500 mono-nucleated cells were counted for each three independent replicates ‘blindly’ using a light microscope. The means of the three counts were represented.

### DNA extraction for epigenetic studies

DNA extraction involved use of the Qiagen DNA/RNA extraction mini kit (QIAGEN, Antwerp, Belgium) following the instructions. The experiment was carried out with three replicates. Untreated and vehicle (dispersion medium)-treated cells were used as a negative control, and 5-Aza-2′-deoxycytidine or in other name, Decitabine (Sigma Aldrich, Brussels, Belgium), a DNA hypomethylation agent, −treated cells (0.1 μM) were used as a positive control. DNA quantification involved use of Nano-drop (Thermo Scientific, 2000c).

### Non-specific DNA methylation and hydroxymethylation analysis by LC-MS/MS

A validated protocol of LC-MS/MS (Waters) was used for identifying and quantifying DNA methylation and hydroxymethylation [22]. In total, 1 μg DNA was spiked with the internal standard mixture and dried. The DNA was enzymatically hydrolyzed to individual deoxyribonucleosides with 10 μl digestion mixture containing phosphodiesteraseI, alkaline phosphatase and benzonase nuclease in Tris-HCl buffer at 37 °C for 12 h. Diluted acetonitrile (Fischer Scientific, UK) was added to each sample. During the procedure, direct light was avoided to minimize potential deamination of target compounds.

### Gene-specific whole-genome DNA methylation microarray and RNA sequencing

An amount of 200 ng genomic DNA was bisulfite-treated with use of the EZ DNA mini kit (Zymo Research, Orange, CA) following the instructions. Genome-wide assessment of DNA methylation involved the Infinium HumanMethylation450 BeadChip Array.

To analyze the effect of methylation on the transcriptome, RNA sequencing was performed. TruSeq RNA access Library Prep Kit (Illumina) was used to prepare the libraries. The resulting libraries were quantified by qPCR with KAPA Library Quantification for Illumina (Kapa Biosystems) and sequenced on a HiSeq2500 (Illumina) by using a V4 flowcell generating 1 × 50 bp reads.

### Data preprocessing and bioinformatics/biostatistical analysis

The Illumina Infinium methylation microarray and RNA-Seq data were processed using Bioconductor R-packages [51].

The ‘minfi’ package was used for quality check and Type I and II probe normalization by the SWAN method [52]. The ‘IMA’ package was used for data annotation and further filtering [53]. After data preprocessing, differentially methylated CpG sites were found by using the ‘limma’ package with a linear modeling approach and empirical Bayes statistics while correcting for batch effect [53, 54]. Gene promoter methylation was defined as the mean methylation level of all CpGs located in a CpG island or shore, between 2000 bp upstream and 500 bp downstream of the gene start site as defined by Ensemble gene annotation v75. Individual CpG sites were linked to genes on the basis of the annotation provided by Illumina. Data were converted into *M* values because they are preferred over *β* values for small sample sizes (e.g., < 10) [55] by the following equation:

$$ \mathrm{M}={\log}_2\left\{\upbeta /\left(1-\upbeta \right)\right\} $$

All analyses were performed with correction for batch effect. Finally, all *p* values were corrected for False Discovery Rate (FDR) [56]. Results were deemed significant with the FDR adjusted *p* < 0.05 (*q* < 0.05).

RNA sequencing reads were processed as previously described [57]. Briefly, the trimmed reads were mapped to the human transcriptome and reference genome (GRCh37.65/hg19) by using TopHat 2.0 [58] and Bowtie 2.0 [59]. Reads were assigned to ensemble gene IDs by using the HTSeq software package. On average, 32,031,451 (+ − 3,819,051) reads were assigned to genes. Differential expression between the different exposure methods was calculated by using EdgeR [60].

### Heatmap and correlation analyses

For DNA methylation and for gene expression dataset the genes were ranked by their FDR corrected *p* values. *Z* scores for the most significant 500 genes for both type of CNTs were plotted on a heat-map. The clustering analysis involved use of Recursively Partitioned Mixture Model in R (RPMM/R) [61]. Representation of the correlation between RNA expression and DNA methylation data was performed using R, smoothscatter function [51].

### Functional GO, pathway and network analysis

Differentially methylated genes that may affect the transcription of genes were proceeded for Gene Ontology (GO, The Gene Ontology Consortium 2000, http://geneontology.org/) analysis, Kyoto Encyclopedia of Genes and Genomes (KEGG, http://www.genome.jp/kegg/) pathway analysis and gene functional classification analysis (for top 3000 genes if data set is > 3000 genes) [62–65]. The web-tool DAVID 6.8 (updated in May 2016) was used in contrast to *Homo sapiens* background [66, 67] at EASE 0.1. When FDR corrected p value was smaller than 0.05, the GO annotation or KEGG pathway were considered significant. For gene functional classification analysis highest stringency was used and top five cluster was selected for interpretation. GeneMania web-tool was used for functional network analysis (http://www.genemania.org) [68].

### Gene expression analysis by RT-PCR

Primer sequences were designed according to online bioinformatics tool, primer bank (https://pga.mgh.harvard.edu/primerbank/index.html) [69–71]. The primers were further justified in the NCBI primer designing tool (https://www.ncbi.nlm.nih.gov/tools/primer-blast/) Ye et al., [72]. The forward and reverse primers of the corresponding gene were listed in Table 1.

**Table 1:** The primer sequences of the tested genes

Primers	
Name	Sequence
BAX forward	CCC GAG AGG TCT TTT TCC GAG
BAX reverse	CCA GCC CAT GAT GGT TCT GAT
TP53 forward	CAG CAC ATG ACG GAG GTT GT
TP53 reverse	TCA TCC AAA TAC TCC ACA CGC
AKT1 forward	GTC ATC GAA CGC ACC TTC CAT
AKT1 reverse	AGC TTC AGG TAC TCA AAC TCG T
NF1 forward	AGA TGA AAC GAT GCT GGT CAA A
NF1 reverse	CCT GTA ACC TGG TAG AAA TGC GA
ATM forward	GGC TAT TCA GTG TGC GAG ACA
ATM reverse	TGG CTC CTT TCG GAT GAT GGA
BCL2L11 forward	TAA GTT CTG AGT GTG ACC GAG A
BCL2L11 reverse	GCT CTG TCT GTA GGG AGG TAG G
GAPDH forward	TGG TAT CGT GGA AGG ACT CA
GAPDH reverse	CCA GTA GAG GCA GGG ATG AT

For the validation assay, we performed four replicates (*N* = 4). The exposures were performed from two different stock solutions on four different T25 vials. RNA is extracted immediately by AllPrep DNA/RNA mini kit (QIAGEN, Belgium) and quality and quantity was obtained using Nano-drop (Thermo Scientific, 2000c). 1 μg of RNA is converted to complementary DNA (cDNA) using SuperScript III First strand kit (Invitrogen, Belgium) according to manual of the kit. After cDNA conversion, gene of interest were amplified using Platinum® SYBR® Green qPCR SuperMix-UDG (Invitrogen, Belgium) according to the kits manual. Westburg Eco 48 well custom reaction plates (Westburg, The Netherlands) and Westburg Eco Adhesive seals (Westburg, The Netherlands) were used to carry out the reverse transcription-PCR (RT-PCR) experiment. GAPDH gene is included as a housekeeping gene. Log fold changes were calculated in relative to the results obtained from vehicle-treated cells. The data was analysed in relative to the vehicle-treated control cells. 2^−∆∆*Ct*^ values were calculated to show the relative fold change of the expression.

### Statistical analysis

Statistical analyses concerning cytotoxicity and genotoxicity involved the use of one way ANOVA with Dunnett’s multiple comparison. The statistics concerning nuclear deposition measurements is conducted using one way ANOVA with Tukey’s multiple comparison. These statistics were performed using graphpad Prism 5 for Windows (GraphPad Software, La Jolla, CA, https://www.graphpad.com/). The statistics for RT-PCR is conducted using two-tailed t-test (interval 95%). Data are represented as mean ± SD. The bioinformatics and statistics for the microarray data were performed by using R explained in the section Data preprocessing and bioinformatics/biostatistics analyses.

## Additional files

Additional file 1: **Figure S1.** Validation of the label-free imaging of CNTs. **Figure S2.** Cytotoxicity assessment of MWCNTs and SWCNTs at a variety of doses. **Figure S3.** DNA damaging effects of MWCNTs and SWCNTs. **Figure S4.** Micronuclei formation in the cells after exposure to MWCNTs and SWCNTs. **Table S1.** Physicochemical characterization of MWCNTs and SWCNTs. **Table S2.** Enriched GO terms by differentially methylated genes (by gene promoter regions) after exposure to MWCNTs. **Table S3.** Gene functional classification analysis using differentially methylated genes (by gene promoter regions) after exposure to MWCNTs. **Table S4.** Enriched GO terms by differentially expressed genes (by gene promoter regions) after exposure to MWCNTs. **Table S5.** Enriched KEGG terms by differentially expressed genes (by gene promoter regions) after exposure to MWCNTs. P53 signalling was noted in italic since its close proximity to significance. **Table S6.** Gene functional classification analysis using differentially expressed genes after exposure to MWCNTs. **Table S7.** Enriched GO terms by differentially methylated genes (by single CpG sites on the genomic regions) after exposure to SWCNTs. **Table S8.** Enriched KEGG terms by differentially methylated genes (by single CpG sites on the genomic regions) after exposure to SWCNTs. **Table S9.** Gene functional classification analysis using differentially methylation CpG sites after exposure to SWCNTs. **Table S10.** Enriched GO terms by differentially expressed genes (by single CpG sites) after exposure to SWCNTs. **Table S11.** Enriched KEGG terms by differentially expressed genes (by single CpG sites) after exposure to SWCNTs. **Table S12.** Gene functional classification analysis using differentially expressed genes after exposure to SWCNTs. (DOCX 596 kb)

Additional file 2: **Video S1.** 3D video of z-stacks images (top to bottom) of MWCNTs in the nucleus. (AVI 42 kb)

Additional file 3: **Video S2.** 3D video of z-stacks images (top to bottom) of SWCNTs in the nucleus. (AVI 28 kb)

Additional file 4: **Video S3.** 3D video of MWCNTs around the nucleus. (AVI 54 kb)

Additional file 5: **Video S4.** 3D video of SWCNTs around the nucleus. (AVI 49 kb)

## Abbreviations

[BCL2L11 (BIM)]: BCL2 like 11
[CDKN1A (p21)]: Cyclin dependent kinase inhibitor 1A
AKAP8L: A-kinase anchoring protein 8 like
ATM: ATM serine/tyrosine kinase
BAX: BCL2 associated x, apoptosis regulator
BCL3: B cell lymphoma 3
CCL2: C-C motif chemokine ligand 2
CCL4: C-C motif chemokine ligand 4
CCL5: C-C motif chemokine ligand 5
CDK4: Cyclin dependent kinase 4
CDK6: Cyclin dependent kinase 6
CDKs: Cyclin dependent kinases
CHK2: Checkpoint kinase 2
CNTs: Carbon nanotubes
CpG: Cytosine-phosphate-guanine
CXCL1: Chemokine (C-X-C motif) ligand 1
CXCL2: Chemokine (C-X-C motif) ligand 2
CytB: Cytochalasin B
DDAH2: Dimethylarginine dimethylaminohydrolase 2
DDR1: Discoidin domain receptor tyrosine kinase 1
DLS: Dynamic light scattering
DMEM: Dulbecco’s Modified Eagle Medium
DMEM/F12: Dulbecco’s Modified Eagle Medium: Nutrient Mixture F12
DNMT1: DNA methyl transferase 1
ECD: Ecdysoneless cell cycle regulator
EIF4E: Eukaryotic translation initiation factor 4E
ENPRA: Engineered Nanoparticle Risk Assessment
ERG: ERG ETS transcription factor
FAS: Fas cell surface death receptor
FCS: Fetal calf serum
FGFR1: Fibroblast growth factor receptor 1
FOXK2: Forkhead box K2
FOXO: Forkhead box O
GO: Gene Ontology
GTP: Guanosine-5′-triphosphate
GTSP1: Glutathione s-transferase pi 1
HBSS: Hank’s Balanced Salt Solution
HTLV-I: Human t-lymphotropic virus-1
IFN-γ: Interferon-gamma
IGFR: Insulin-like growth factor 1 receptor
IL1-β: Interleukin 1 beta
IL6: Interleukin 6
KEGG: Kyoto Encyclopedia of Genes and Genomes
KIF15: Kinesin family member 15
KLF2: Kruppel like factor 2
LC-MS/MS: Liquid chromatography-mass spectrometry
LDH: Lactate dehydrogenase
LIF: Leukemia inhibitory factor
MAPK: Mitogen-activated protein kinase
MLKL: Mixed lineage kinase domain like
MNs: Micronuclei
MWCNTs: Multi-walled CNTs
NF1: Neurofibromatosis type I
Nm: Nanometer
NOSTRIN: Nitric oxide synthase trafficking
NOXA1: NADPH oxidase activator 1
NF-κB: Nuclear factor-kappa B
NFKBIA: Nuclear factor-kappa B inhibitor alpha
PBS: Phosphate buffered saline
PDGFR: Platelet-derived growth factor receptor beta
PERP: PERP TP53 apoptosis effector
PI3K-AKT: Phosphatidylinositide 3 kinases-protein kinase B
PIK3R2: Phosphoinositide-3-kinase regulatory subunit 2
RTKs: Receptor tyrosine kinases
SHISA5: Shisa family member 5
SHROOM2: Shroom family member 2
SKI: SKI proto-oncogene
snoU13: Small nucleolar RNA U13
SWCNTs: Single-walled CNTs
TEM: Transmission electron microscopy
TNF: Tumour necrosis factor
TNF-α: Tumour necrosis factor alpha
TNFRSF25: Tumour necrosis factor receptor superfamily member 25
TP53: Tumour protein 53
TP53I3: Tumour protein P53 inducible protein 3
TRAF2: Tumour necrosis factor receptor associated factor 2
UDP: Uridine 5′-diphospho-glucuronosyltransferase
VHL: Von hippel-lindau tumor suppressor
WST-1: Water-soluble tetrazolium salt-1
5-mC: 5-methylcytosine
5-hmC: 5-hydroxymethylcytosine
