# Supplementary material for: Differences in MWCNT- and SWCNT-induced DNA methylation alterations in association with the nuclear deposition
Source: Part Fibre Toxicol. 2018 Feb 9;15:11. doi: 10.1186/s12989-018-0244-6 (PMC5807760; doi:10.1186/s12989-018-0244-6)
Supplement: Supplementary file 1 — Figure S1. Validation of the label-free imaging of CNTs. Figure S2. Cytotoxicity assessment of MWCNTs and SWCNTs at a variety of doses. Figure S3. DNA damaging effects of MWCNTs and SWCNTs. Figure S4. Micronuclei formation in the cells after exposure to MWCNTs and SWCNTs. Table S1. Physicochemical characterization of MWCNTs and SWCNTs. Table S2. Enriched GO terms by differentially methylated genes (by gene promoter regions) after exposure to MWCNTs. Table S3. Gene functional classification analysis using differentially methylated genes (by gene promoter regions) after exposure to MWCNTs. Table S4. Enriched GO terms by differentially expressed genes (by gene promoter regions) after exposure to MWCNTs. Table S5. Enriched KEGG terms by differentially expressed genes (by gene promoter regions) after exposure to MWCNTs. P53 signalling was noted in italic since its close proximity to significance. Table S6. Gene functional classification analysis using differentially expressed genes after exposure to MWCNTs. Table S7. Enriched GO terms by differentially methylated genes (by single CpG sites on the genomic regions) after exposure to SWCNTs. Table S8. Enriched KEGG terms by differentially methylated genes (by single CpG sites on the genomic regions) after exposure to SWCNTs. Table S9. Gene functional classification analysis using differentially methylation CpG sites after exposure to SWCNTs. Table S10. Enriched GO terms by differentially expressed genes (by single CpG sites) after exposure to SWCNTs. Table S11. Enriched KEGG terms by differentially expressed genes (by single CpG sites) after exposure to SWCNTs. Table S12. Gene functional classification analysis using differentially expressed genes after exposure to SWCNTs. (DOCX 596 kb) [file 12989_2018_244_MOESM1_ESM.docx]

# **Differences in MWcnt- and swCNT-induced DNA methylation alterations in association with the NUCLEAR DEPOSITION SUPPLEMENTARY iNFORMATION**

Deniz Öner^1#^, Manosij Ghosh^1#^, Hannelore Bové^2,3^, Matthieu Moisse^4,5^, Bram Boeckx^4,5^, Radu C Duca^6^, Katrien Poels^6^, Katrien Luyts^1^, Eveline Putzeys^1,7^, Kirsten Van Landuydt^7^, Jeroen Vanoirbeek^1,6^, Marcel Ameloot^3^, Diether Lambrechts^4,5^, Lode Godderis^6,8^, and Peter HM Hoet^1^

*^1^Laboratory of Toxicology, Unit of Environment and Health, Department of Public Health and Primary Care, KU Leuven, 3000 Leuven, Belgium*

*^2^Centre for Surface Chemistry and Catalysis, Celestijnenlaan 200F, KU Leuven, 3001 Leuven, Belgium*

*^3^Biomedical Research Institute, Agoralaan Building C, Hasselt University, 3590 Diepenbeek, Belgium*

*^4^Laboratory for Translational Genetics, Department of Human Genetics, KU Leuven, 3000 Leuven, Belgium*

*^5^Laboratory for Translational Genetics, VIB Centre for Cancer Biology, VIB, 3000 Leuven, Belgium*

*^6^ Laboratory for Occupational and Environmental Hygiene, Unit of Environment and Health, Department of Public Health and Primary Care, KU Leuven, 3000 Leuven, Belgium*

*^7^Department of Oral Health Sciences, Unit of Biomaterials (BIOMAT), KU Leuven, 3000 Leuven, Belgium*

*^8^IDEWE, External Service for Prevention and Protection at Work, B-3001, 3000 Leuven, Belgium*

# DÖ and MG have equal contribution

Corresponding author: [peter.hoet@kuleuven.be](mailto:peter.hoet@kuleuven.be)

## Figures


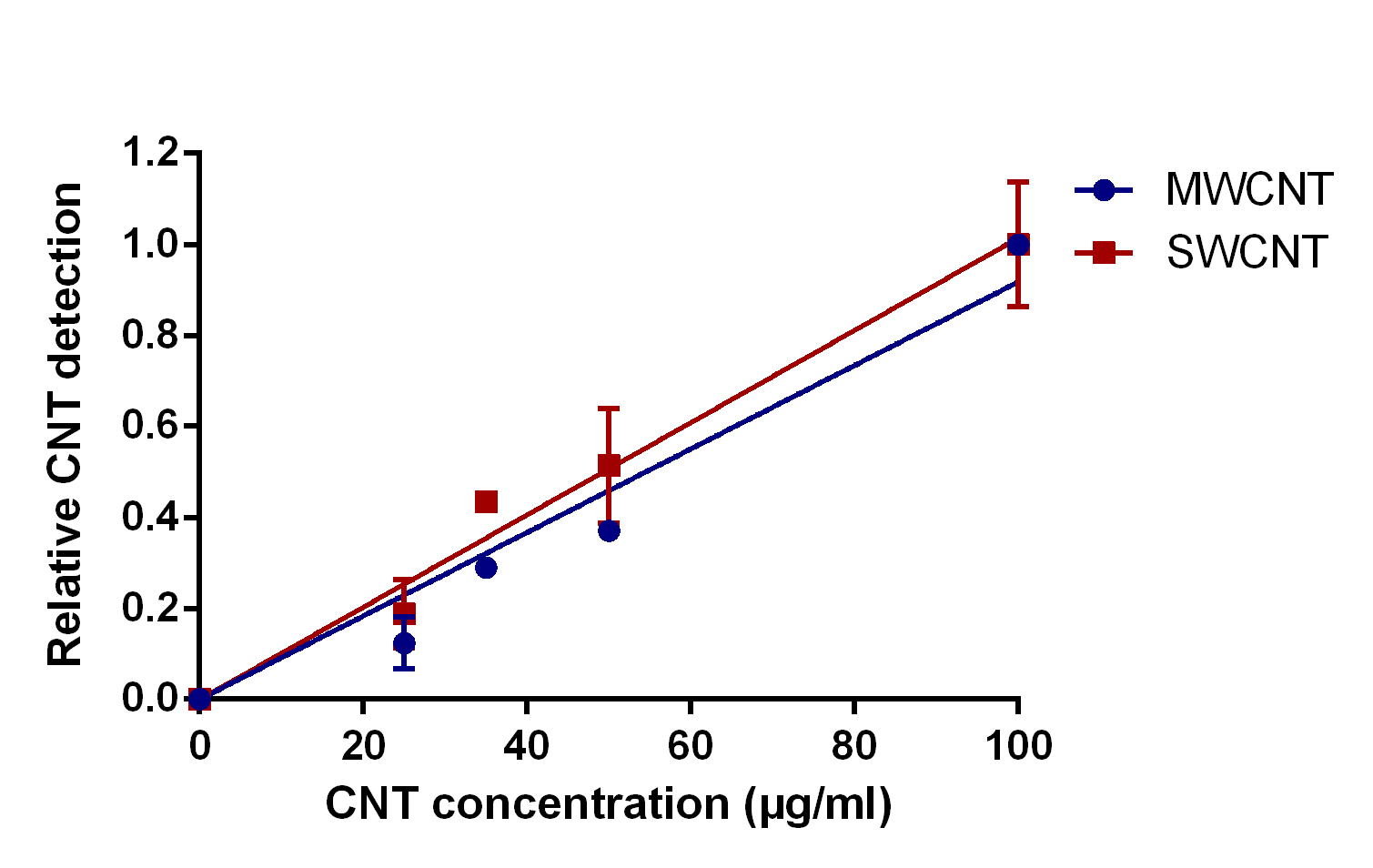


**S. figure 1**: Validation of the label-free imaging of CNTs.

A linear relation between actual and detected number of CNTs was observed for MWCNTs and SWCNTs when spiked in ultrapure water. The amount of detected CNTs is expressed relatively to the detected number at the highest concentration ($N$ = 6). The regression coefficient $R^{2}$ was 0.95 for MWCNT and 0.92 for SWCNT.


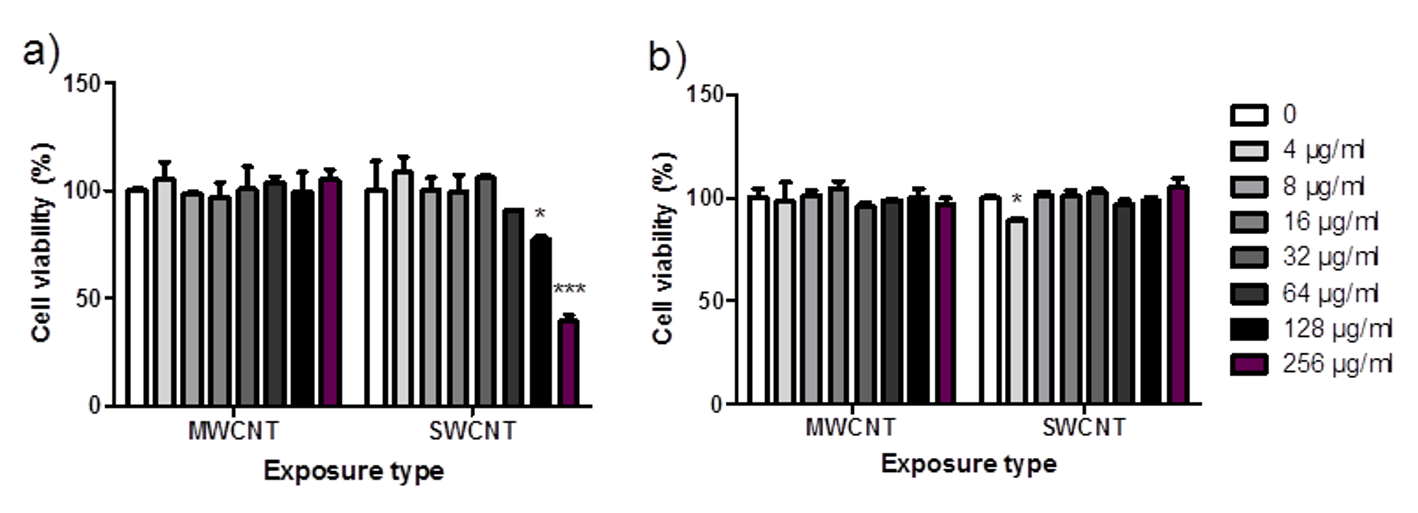


**S. figure 2**: Cytotoxicity assessment of MWCNTs and SWCNTs at a variety of doses. Cytotoxicity was measured after exposure to MWCNTs and SWCNT at various doses (4, 8, 16, 32, 128 and 256 µg/ml) on human bronchial cells (16HBE) in comparison with untreated cells. Two different assays, namely WST-1 and lactate dehydrogenase (LDH) assays, were performed. The doses are indicated in the legend and the relative (%) viability is represented on the y-axis. a) Cellular viability of the cells after 24 h exposure to MWCNTs and SWCNTs measured by WST-1 assay. b) Cellular viability of the cells after 24 h exposure to MWCNTs and SWCNTs measured by LDH assay.* indicates p < 0.05, ** indicates p < 0.01 and *** indicates p 0.001. (N=2, One way ANOVA with Dunnett’s multiple comparison)


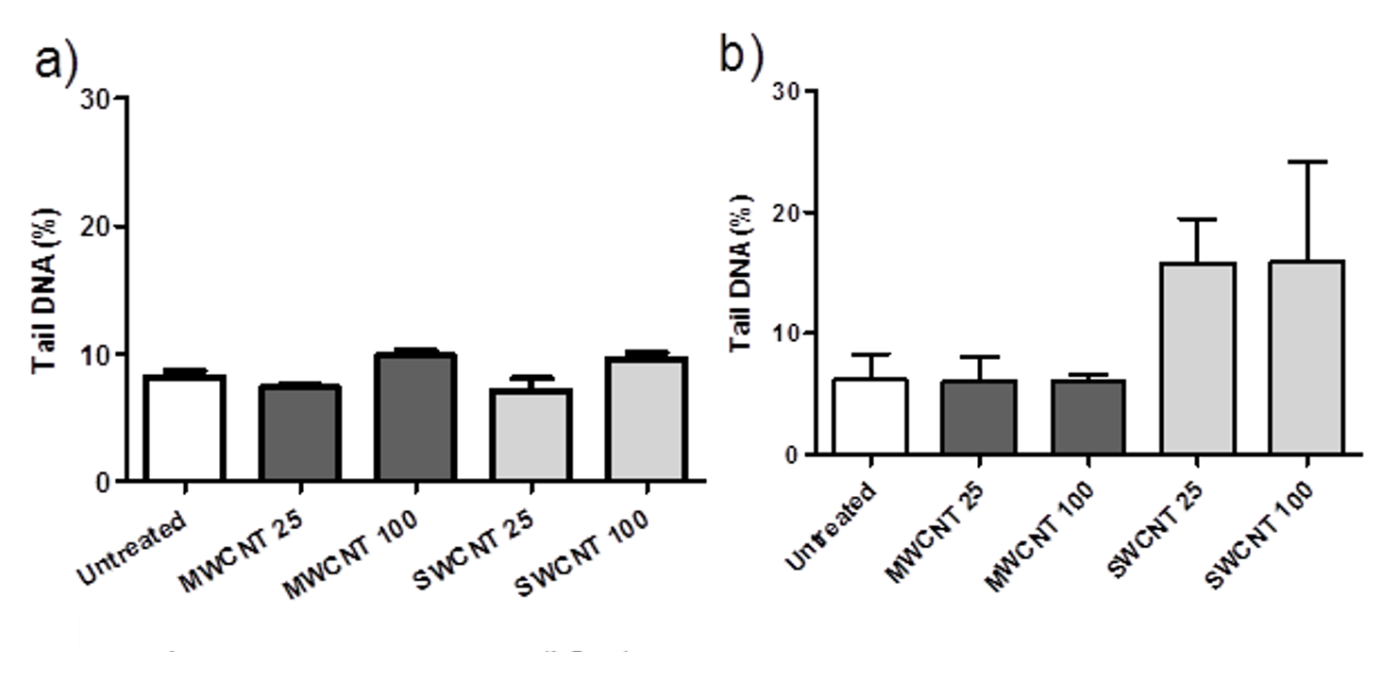


**S. figure 3**: DNA damaging effects of MWCNTs and SWCNTs. DNA damage (strand breaks) was measured by alkaline comet assay after exposure to 25 and 100 µg/ml of MWCNTs and SWCNTs for (a) 3 h and for (b) 24 h. DNA Tail (%) was demonstrated on the y-axis. Treatment conditions are indicated on the x-axis. The data obtained from MWCNTs-exposed cells are shown in dark gray, SWCNTs-exposed cells are shown in light gray, untreated cells were shown in white color. No significant increase in DNA damage was noted in comparison to untreated controls. (N=2, One way ANOVA, Dunnett’s multiple comparison).


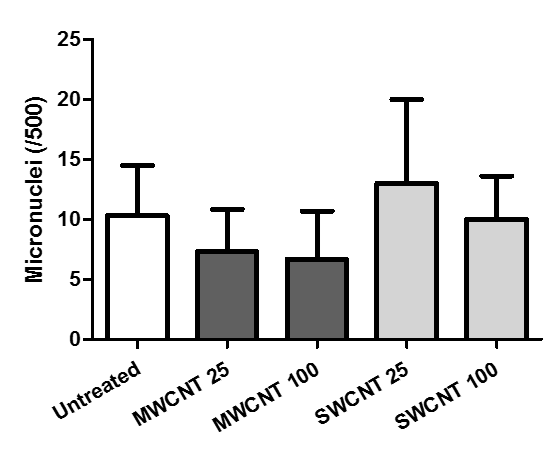


**S. figure 3**: Micronuclei formation in the cells after exposure to MWCNTs and SWCNTs. Micronuclei formation measured by micronucleus assay after exposure to 25 and 100 µg/ml of MWCNTs and SWCNTs. Number of micronuclei was counted in five hundred cells and represented on the y-axis. Treatment conditions are represented on the x-axis. The data obtained from MWCNTs-exposed cells are shown in dark gray, SWCNTs-exposed cells are shown in light gray and untreated cells were shown in white color. No statistical significance was detected.

## Tables

|  | **MWCNTs** | **SWCNTs** |
| --- | --- | --- |
| **Dimensions** | length: 846 ± 446 nm,  diameter: 11 ± 3 nm surface area(BET): 254.00(m^2^/g) | diameter: 0.8 nm  pristine length: ~ 8000 nm |
| **Metal impurities** | trace metals: Al, Fe, Na, S, Co, Ca, K | trace metals: Al, As, B, Ba, Ca, Cl, Co, Cs, Cu, Dy, Eu, Gd, Hf, La, Mg, Mn, Mo, Na, Sm, Th, V and W. |
| **Endotoxin contamination** | Not detected. | Not detected. |
| **DLS measurements** | Stable dispersion and diameter. | Stable, increased average size-mass by SWCNT 100. |

**S. table 1**: Physicochemical characterization of MWCNTs and SWCNTs.

The reference properties were included (aspect ratio and metal impurities) referred to NIST and JRC[1,2]. Further characterizations (endotoxin contamination and DLS measurements) were conducted in experimental conditions.

| **GO number** | **GO Term** | **Count** | **FDR corrected *p* value** |
| --- | --- | --- | --- |
| GO:0007275 | Multicellular organism development | 77 | 1.3E-2 |
| GO:0051056 | Regulation of small GTPase mediated signal transduction | 29 | 2.3E-2 |

**S. table 2**: Enriched GO terms by differentially methylated genes (by gene promoter regions) after exposure to MWCNTs.

In s. tables 2, 4-5, 7-8, 10-11, ‘Count’ indicates the number of genes of differentially methylated genes within the corresponding GO.

| **Gene Group 1** | **Enrichment Score: 2.74** | **Gene Name** |
| --- | --- | --- |
| 1 | T-cell leukemia homeobox 2 | *TLX2* |
| 2 | NK6 homeobox 3 | *NKX6-3* |
| 3 | T-cell leukemia homeobox 1 | *TLX1* |
| 4 | homeobox B6 | *HOXB6* |
| 5 | homeobox B4 | *HOXB4* |
| 6 | homeobox B3 | *HOXB3* |
| 7 | homeobox D4 | *HOXD4* |
| 8 | BarH like homeobox 2 | *BARHL2* |
| 9 | homeobox D3 | *HOXD3* |
| 10 | even-skipped homeobox 1 | *EVX1* |
| 11 | homeobox A10 | *HOXA10* |
| 12 | homeobox C11 | *HOXC11* |
| 13 | homeobox A7 | *HOXA7* |
| 14 | tetrapeptide repeat homeobox 1 | *TPRX1* |
| 15 | homeobox C6 | *HOXC6* |
| 16 | homeobox A3 | *HOXA3* |
| 17 | distal-less homeobox 6 | *DLX6* |
| **Gene Group 2** | **Enrichment Score: 2.67** |  |
| 1 | ArfGAP with coiled-coil, ankyrin repeat and PH domains 3 | *ACAP3* |
| 2 | ArfGAP with GTPase domain, ankyrin repeat and PH domain 3 | *AGAP3* |
| 3 | GIT ArfGAP 1 | *GIT1* |
| 4 | ArfGAP with SH3 domain, ankyrin repeat and PH domain 3 | *ASAP3* |
| 5 | ArfGAP with dual PH domains 1 | *ADAP1* |
| **Gene Group 3** | **Enrichment Score: 2.53** |  |
| 1 | mitogen-activated protein kinase kinase kinase kinase 1 | *MAP4K1* |
| 2 | casein kinase 1 gamma 2 | *CSNK1G2* |
| 3 | pregnancy up-regulated nonubiquitous CaM kinase | *PNCK* |
| 4 | mitogen-activated protein kinase kinase kinase 6 | *MAP3K6* |
| 5 | apoptosis associated tyrosine kinase | *AATK* |
| 6 | BR serine/threonine kinase 1 | *BRSK1* |
| 7 | WNK lysine deficient protein kinase 2 | *WNK2* |
| 8 | dual specificity tyrosine phosphorylation regulated kinase 1B | *DYRK1B* |
| 9 | WNK lysine deficient protein kinase 4 | *WNK4* |
| 10 | calcium/calmodulin dependent protein kinase kinase 2 | *CAMKK2* |
| 11 | CDC like kinase 2 | *CLK2* |
| 12 | protein kinase, membrane associated tyrosine/threonine 1 | *PKMYT1* |
| 13 | SH3 domain binding kinase family member 2 | *SBK2* |
| 14 | serine/threonine kinase 25 | *STK25* |
| 15 | dual specificity tyrosine phosphorylation regulated kinase 4 | *DYRK4* |
| 16 | lemur tyrosine kinase 3 | *LMTK3* |
| 17 | cyclin dependent kinase 18 | *CDK18* |
| 18 | MAP kinase interacting serine/threonine kinase 2 | *MKNK2* |
| 19 | tousled like kinase 1 | *TLK1* |
| **Gene Group 4** | **Enrichment Score: 2.53** |  |
| 1 | protein phosphatase 1 regulatory subunit 12C | *PPP1R12C* |
| 2 | cortactin binding protein 2 | *CTTNBP2* |
| 3 | ankyrin repeat domain 20 family member A3 | *ANKRD20A3* |
| 4 | POTE ankyrin domain family member F | *POTEF* |
| 5 | ankyrin repeat domain 20 family member A2 | *ANKRD20A2* |
| **Gene Group 5** | **Enrichment Score: 2.17** |  |
| 1 | platelet endothelial aggregation receptor 1 | *PEAR1* |
| 2 | scavenger receptor class F member 1 | *SCARF1* |
| 3 | delta/notch like EGF repeat containing | *DNER* |
| 4 | multiple EGF like domains 11 | *MEGF11* |
| 5 | scavenger receptor class F member 2 | *SCARF2* |
| 6 | stabilin 1 | *STAB1* |

**S. table 3**: Gene functional classification analysis using differentially methylated genes (by gene promoter regions) after exposure to MWCNTs. Gene group number (ranked by the enrichment score), the enrichment score for each gene group and official gene names were written on the headers of the each subgroup.

| **GO number** | **GO Term** | **Count** | **FDR corrected *p* value** |
| --- | --- | --- | --- |
| **GO:0006886** | **Intracellular protein transport** | **80** | **1.2E-3** |
| GO:0006468 | Protein phosphorylation | 134 | 2.3E-3 |

**S. table 4**: Enriched GO terms by differentially expressed genes (by gene promoter regions) after exposure to MWCNTs. The overlapped (shared) GOs and pathways between table 4 vs. 10 and table 5 vs. 11 are indicated in bold.

| **KEGG number** | **KEGG Term** | **Count** | **FDR corrected *p* value** | |
| --- | --- | --- | --- | --- |
| **hsa01100** | **Metabolic pathways** | **295** | **7.2E-3** |  |
| hsa05166 | HTLV-I infection | 79 | 1.2E-2 |  |
| *hsa04115* | *p53 signaling pathway* | *28* | *7.3E-2* |  |

**S. table 5**: Enriched KEGG terms by differentially expressed genes (by gene promoter regions) after exposure to MWCNTs. P53 signalling was noted in italic since its close proximity to significance.

| **Gene Group 1** | **Enrichment Score: 16.84** | **Gene Name** |
| --- | --- | --- |
| 1 | deoxynucleotidyltransferase terminal interacting protein 1 | *DNTTIP1* |
| 2 | CGG triplet repeat binding protein 1 | *CGGBP1* |
| 3 | TSC22 domain family member 4 | *TSC22D4* |
| 4 | DR1 associated protein 1 | *DRAP1* |
| 5 | GC-rich promoter binding protein 1 | *GPBP1* |
| 6 | transcription factor 25 | *TCF25* |
| 7 | ligand dependent nuclear receptor corepressor | *LCOR* |
| **Gene Group 2** | **Enrichment Score: 14.06** |  |
| 1 | Cbp/p300 interacting transactivator with Glu/Asp rich carboxy-terminal domain 4 | *CITED4* |
| 2 | SERTA domain containing 2 | *SERTAD2* |
| 3 | proline rich 13 | *PRR13* |
| 4 | COMM domain containing 2 | *COMMD2* |
| 5 | COMM domain containing 4 | *COMMD4* |
| 6 | GC-rich promoter binding protein 1 | *GPBP1* |
| 7 | COMM domain containing 5 | *COMMD5* |
| 8 | proline rich nuclear receptor coactivator 1 | *PNRC1* |
| 9 | PPARGC1 and ESRR induced regulator, muscle 1 | *PERM1* |
| 10 | vestigial like family member 2 | *VGLL2* |
| 11 | COMM domain containing 9 | *COMMD9* |
| **Gene Group 3** | **Enrichment Score: 13.77** |  |
| 1 | ring finger protein 126 | *RNF126* |
| 2 | mahogunin ring finger 1 | *MGRN1* |
| 3 | ring finger protein 11 | *RNF11* |
| 4 | ring finger protein 182 | *RNF182* |
| 5 | ring finger protein 25 | *RNF25* |
| 6 | ring finger protein 187 | *RNF187* |
| 7 | neuralized E3 ubiquitin protein ligase 1B | *NEURL1B* |
| 8 | deltex E3 ubiquitin ligase 2 | *DTX2* |
| 9 | ring finger protein 220 | *RNF220* |
| 10 | neuralized E3 ubiquitin protein ligase 3 | *NEURL3* |
| 11 | ring finger and CHY zinc finger domain containing 1 | *RCHY1* |
| **Gene Group 4** | **Enrichment Score: 12.37** |  |
| 1 | NSL1, MIS12 kinetochore complex component | *NSL1* |
| 2 | centromere protein T | *CENPT* |
| 3 | NUF2, NDC80 kinetochore complex component | *NUF2* |
| 4 | centromere protein O | *CENPO* |
| 5 | zwilch kinetochore protein | *ZWILCH* |
| 6 | kinetochore associated 1 | *KNTC1* |
| **Gene Group 5** | **Enrichment Score: 11.72** |  |
| 1 | CORO7-PAM16 readthrough | *CORO7-PAM16* |
| 2 | striatin 4 | *STRN4* |
| 3 | PWP2 periodic tryptophan protein homolog (yeast) | *PWP2* |
| 4 | F-box and WD repeat domain containing 9 | *FBXW9* |
| 5 | WD repeat domain 43 | *WDR43* |
| 6 | glutamate rich WD repeat containing 1 | *GRWD1* |
| 7 | WD repeat containing, antisense to TP73 | *WRAP73* |
| 8 | transducin beta like 3 | *TBL3* |
| 9 | diphthamide biosynthesis 7 | *DPH7* |
| 10 | WD repeat domain 18 | *WDR18* |
| 11 | bromodomain and WD repeat domain containing 3 | *BRWD3* |
| 12 | WD repeat domain 77 | *WDR77* |
| 13 | WD repeat domain 90 | *WDR90* |
| 14 | WD repeat domain 34 | *WDR34* |
| 15 | DDB1 and CUL4 associated factor 10 | *DCAF10* |
| 16 | echinoderm microtubule associated protein like 3 | *EML3* |
| 17 | WD repeat domain 13 | *WDR13* |
| 18 | tumor suppressing subtransferable candidate 1 | *TSSC1* |
| 19 | WD repeat domain 4 | *WDR4* |
| 20 | SH3KBP1 binding protein 1 | *SHKBP1* |
| 21 | MTOR associated protein, LST8 homolog | *MLST8* |
| 22 | ribosomal RNA processing 9, small subunit (SSU) processome component, homolog (yeast) | *RRP9* |
| 23 | WD repeat domain 46 | *WDR46* |
| 24 | Dmx like 1 | *DMXL1* |
| 25 | coronin 6 | *CORO6* |
| 26 | POC1 centriolar protein B | *POC1B* |
| 27 | DDB1 and CUL4 associated factor 4 | *DCAF4* |
| 28 | zinc finger protein 106 | *ZNF106* |
| 29 | WD repeat domain 88 | *WDR88* |
| 30 | WD repeat domain 62 | *WDR62* |
| 31 | WD repeat domain 26 | *WDR26* |
| 32 | notchless homolog 1 | *NLE1* |
| 33 | WD repeat domain 74 | *WDR74* |
| 34 | Dmx like 2 | *DMXL2* |

***S. table 6****: Gene functional classification analysis using differentially expressed genes after exposure to MWCNTs*

| **GO number** | **GO Term** | **Count** | **FDR corrected *p* value** |
| --- | --- | --- | --- |
| GO: 0052697 | Xenobiotic glucuronidation | 8 | 1.6E-7 |
| GO: 2001030 | Negative regulation of glucuronosyltransferase activity | 7 | 5.6E-6 |
| GO: 1904224 | Negative regulation of cellular glucuronidation | 7 | 5.6E-6 |
| GO: 0045922 | Negative regulation of fatty acid metabolic process | 7 | 1.6E-5 |
| GO: 0052696 | Flavonoid glucuronidation | 8 | 5.9E-4 |
| GO: 0052695 | Cellular glucuronidation | 7 | 1.4E-3 |

**S. table 7**: Enriched GO terms by differentially methylated genes (by single CpG sites on the genomic regions) after exposure to SWCNTs.

| **KEGG number** | **KEGG Pathway** | **Count** | **FDR corrected *p* value** |
| --- | --- | --- | --- |
| hsa00053 | Ascorbate and aldarate metabolism | 8 | 4.1E-3 |
| hsa00140 | Steroid hormone biosynthesis | 10 | 1.5E-2 |
| hsa00980 | Metabolism of xenobiotics by cytochrome P450 | 11 | 1.7E-2 |
| Hsa00040 | Pentose and glucuronate interconversions | 8 | 3.2E-2 |

**S. table 8**: Enriched KEGG terms by differentially methylated genes (by single CpG sites on the genomic regions) after exposure to SWCNTs.

| **Gene Group 1** | **Enrichment Score: 4.45** | **Gene Name** |
| --- | --- | --- |
| 1 | UDP glucuronosyltransferase family 1 member A4 | *UGT1A4* |
| 2 | UDP glucuronosyltransferase family 1 member A5 | *UGT1A5* |
| 3 | UDP glucuronosyltransferase family 1 member A9 | *UGT1A9* |
| 4 | UDP glucuronosyltransferase family 1 member A6 | *UGT1A6* |
| 5 | UDP glucuronosyltransferase family 1 member A7 | *UGT1A7* |
| 6 | UDP glucuronosyltransferase family 1 member A8 | *UGT1A8* |
| 7 | UDP glucuronosyltransferase family 1 member A10 | *UGT1A10* |
| 8 | UDP glucuronosyltransferase family 1 member A3 | *UGT1A3* |
| **Gene Group 2** | **Enrichment Score: 2.18** |  |
| 1 | androgen induced 1 | *AIG1* |
| 2 | transmembrane protein 181 | *TMEM181* |
| 3 | transmembrane protein 179 | *TMEM179* |
| 4 | family with sequence similarity 189 member A1 | *FAM189A1* |
| 5 | GRAM domain containing 1B | *GRAMD1B* |
| 6 | transmembrane protein 87B | *TMEM87B* |
| **Gene Group 3** | **Enrichment Score: 1.3** |  |
| 1 | zinc finger protein 451 | *ZNF451* |
| 2 | zinc finger protein 146 | *ZNF146* |
| 3 | zinc finger and BTB domain containing 45 | *ZBTB45* |
| 4 | human immunodeficiency virus type I enhancer binding protein 1 | *HIVEP1* |
| 5 | ZFP64 zinc finger protein | *ZFP64* |
| 6 | zinc finger and BTB domain containing 4 | *ZBTB4* |
| 7 | zinc finger protein 286B | *ZNF286B* |
| 8 | GLIS family zinc finger 1 | *GLIS1* |
| 9 | ras responsive element binding protein 1 | *RREB1* |
| 10 | zinc finger protein 350 | *ZNF350* |
| 11 | zinc finger and BTB domain containing 34 | *ZBTB34* |
| 12 | zinc finger protein 354A | *ZNF354A* |
| 13 | zinc finger protein 618 | *ZNF618* |
| 14 | GLIS family zinc finger 3 | *GLIS3* |
| 15 | zinc finger protein 502 | *ZNF502* |
| 16 | zinc finger and SCAN domain containing 5B | *ZSCAN5B* |
| 17 | CCCTC-binding factor like | *CTCFL* |
| 18 | zinc finger protein 827 | *ZNF827* |
| 19 | zinc finger protein 233 | *ZNF233* |
| 20 | zinc finger protein 154 | *ZNF154* |
| 21 | zinc finger protein 20 | *ZNF20* |
| **Gene Group 4** | **Enrichment Score: 0.11** |  |
| 1 | carcinoembryonic antigen related cell adhesion molecule 16 | *CEACAM16* |
| 2 | chromosome 1 open reading frame 204 | *C1orf204* |
| 3 | V-set and immunoglobulin domain containing 8 | *VSIG8* |
| 4 | cell adhesion molecule 4 | *CADM4* |
| 5 | microfibrillar associated protein 3 like | *MFAP3L* |
| 6 | TAP binding protein like | *TAPBPL* |

***S. table 9****: Gene functional classification analysis using differentially methylation CpG sites after exposure to SWCNTs.*

| **GO number** | **GO Term** | **Count** | **FDR corrected *p* value** |
| --- | --- | --- | --- |
| GO:0015031 | protein transport | 146 | 7.5E-5 |
| **GO:0006886** | **intracellular protein transport** | **92** | **2.3E-3** |
| GO:0006977 | DNA damage response, signal transduction by p53 class mediator resulting in cell cycle arrest | *33* | *8.0E-3* |
| GO:0016477 | Cell migration | 70 | 9.0E-3 |
| GO:0006888 | ER to Golgi vesicle mediated transport | 66 | 1.0E-2 |
| GO:0007155 | Cell-cell adhesion | 100 | 1.3E-2 |
| GO:0006914 | Autophagy | 56 | 2.9E-2 |
| GO:0032091 | Negative regulation of protein binding | 30 | 3.2E-2 |

**S. table 10**: Enriched GO terms by differentially expressed genes (by single CpG sites) after exposure to SWCNTs.

| **KEGG number** | **KEGG Term** | **Count** | **FDR corrected *p* value** |
| --- | --- | --- | --- |
| **hsa01100** | **Metabolic pathways** | **375** | **1.3E-5** |
| hsa04144 | Endocytosis | 95 | 5.4E-3 |
| hsa04115 | P53 signalling pathway | 34 | 8.3E-3 |
| hsa01230 | Biosynthesis of antibiotics | 78 | 4.5E-3 |
| *hsa04110* | *Cell cycle* | *48* | *5.0E-2* |

**S. table 11**: Enriched KEGG terms by differentially expressed genes (by single CpG sites) after exposure to SWCNTs. Cell cycle was noted in italic since its close proximity to significance.

| **Gene Group 1** | **Enrichment Score: 11.04** | **Gene Name** |
| --- | --- | --- |
| 1 | transformer 2 alpha homolog | *TRA2A* |
| 2 | RNA binding motif protein 19 | *RBM19* |
| 3 | polypyrimidine tract binding protein 2 | *PTBP2* |
| 4 | epithelial splicing regulatory protein 2 | *ESRP2* |
| 5 | heterogeneous nuclear ribonucleoprotein A/B | *HNRNPAB* |
| 6 | RNA binding motif protein 42 | *RBM42* |
| 7 | splicing factor 3b subunit 4 | *SF3B4* |
| 8 | ribonucleoprotein, PTB binding 1 | *RAVER1* |
| 9 | ribonucleoprotein, PTB binding 2 | *RAVER2* |
| 10 | musashi RNA binding protein 1 | *MSI1* |
| **Gene Group 2** | **Enrichment Score: 10.53** |  |
| 1 | centromere protein T | *CENPT* |
| 2 | SPC25, NDC80 kinetochore complex component | *SPC25* |
| 3 | centromere protein H | *CENPH* |
| 4 | zwilch kinetochore protein | *ZWILCH* |
| 5 | spindle and kinetochore associated complex subunit 2 | *SKA2* |
| 6 | NSL1, MIS12 kinetochore complex component | *NSL1* |
| 7 | DSN1 homolog, MIS12 kinetochore complex component | *DSN1* |
| 8 | centromere protein O | *CENPO* |
| 9 | centromere protein K | *CENPK* |
| 10 | NUF2, NDC80 kinetochore complex component | *NUF2* |
| **Gene Group 3** | **Enrichment Score: 10.44** |  |
| 1 | ankyrin repeat domain 9 | *ANKRD9* |
| 2 | protein phosphatase 1 regulatory subunit 12C | *PPP1R12C* |
| 3 | ankyrin repeat domain 33B | *ANKRD33B* |
| 4 | CASK interacting protein 2 | *CASKIN2* |
| 5 | KN motif and ankyrin repeat domains 4 | *KANK4* |
| 6 | ankyrin repeat domain 46 | *ANKRD46* |
| 7 | osteoclast stimulating factor 1 | *OSTF1* |
| 8 | ankyrin repeat domain 34B | *ANKRD34B* |
| **Gene Group 4** | **Enrichment Score: 9.78** |  |
| 1 | PPARGC1 and ESRR induced regulator, muscle 1 | *PERM1* |
| 2 | proline rich 13 | *PRR13* |
| 3 | COMM domain containing 5 | *COMMD5* |
| 4 | COMM domain containing 4 | *COMMD4* |
| 5 | GC-rich promoter binding protein 1 | *GPBP1* |
| 6 | proline rich nuclear receptor coactivator 1 | *PNRC1* |
| **Gene Group 5** | **Enrichment Score: 9.77** |  |
| 1 | cyclin dependent kinase 18 | *CDK18* |
| 2 | ribosomal protein S6 kinase like 1 | *RPS6KL1* |
| 3 | PAS domain containing serine/threonine kinase | *PASK* |
| 4 | mitogen-activated protein kinase kinase kinase 8 | *MAP3K8* |
| 5 | mitogen-activated protein kinase 6 | *MAPK6* |
| 6 | cyclin dependent kinase 15 | *CDK15* |
| 7 | testis-specific kinase 1 | *TESK1* |
| 8 | mitogen-activated protein kinase-activated protein kinase 3 | *MAPKAPK3* |
| 9 | cyclin dependent kinase like 1 | *CDKL1* |
| 10 | testis-specific kinase 2 | *TESK2* |
| 11 | protein kinase, membrane associated tyrosine/threonine 1 | *PKMYT1* |
| 12 | cyclin dependent kinase like 3 | *CDKL3* |
| 13 | CDC like kinase 2 | *CLK2* |
| 14 | mitogen-activated protein kinase kinase kinase 13 | *MAP3K13* |
| 15 | TRAF2 and NCK interacting kinase | *TNIK* |
| 16 | TTK protein kinase | *TTK* |
| 17 | serine/threonine kinase 10 | *STK10* |
| 18 | aarF domain containing kinase 1 | *ADCK1* |
| 19 | serine/threonine kinase 17b | *STK17B* |
| 20 | TAO kinase 3 | *TAOK3* |
| 21 | receptor interacting serine/threonine kinase 4 | *RIPK4* |
| 22 | serine/threonine kinase 25 | *STK25* |
| 23 | mitogen-activated protein kinase kinase kinase kinase 4 | *MAP4K4* |
| 24 | tau tubulin kinase 2 | *TTBK2* |
| 25 | RIO kinase 3 | *RIOK3* |
| 26 | mitogen-activated protein kinase kinase kinase 6 | *MAP3K6* |
| 27 | casein kinase 1 gamma 3 | *CSNK1G3* |
| 28 | protein kinase D3 | *PRKD3* |
| 29 | ribosomal protein S6 kinase B2 | *RPS6KB2* |
| 30 | G protein-coupled receptor kinase 6 | *GRK6* |
| 31 | protein kinase N3 | *PKN3* |
| 32 | PDZ binding kinase | *PBK* |
| 33 | NIMA related kinase 7 | *NEK7* |
| 34 | WNK lysine deficient protein kinase 2 | *WNK2* |
| 35 | WNK lysine deficient protein kinase 4 | *WNK4* |
| 36 | microtubule associated serine/threonine kinase like | *MASTL* |
| 37 | NIMA related kinase 1 | *NEK1* |

**S. table 12**: Gene functional classification analysis using differentially expressed genes after exposure to SWCNTs.

## Dose specific analysis of the epigenetic data

The differentially methylated CpG sites and gene promoters were investigated on the samples exposed to MWCNTs and SWCNTs with different doses. Exposure to 25 µg/ml of MWCNTs did not induce significant DNA methylation changes, both at CpG sites and gene promoter regions. Exposure to 100 µg/ml of MWCNTs resulted in hypermethylation at one CpG site ($q$ = 0.003, $\Delta\beta$ = 0.170. This hypermethylated CpG site resides on TSS1500 region of *GLRX* gene and acts as an enhancer. At gene promoter level significant hypomethylation ($q$ = 0.02 and $\Delta\beta$ = -0.04) at long intergenic non-protein coding RNA 963 (*LINC00963*) gene was noted.

Exposure to 25 µg/ml of SWCNTs induced significant methylation at two different CpG sites (CpG1 and CpG 2). CpG1 was hypomethylated and resides on a CpG island and at the 1st exon of the *TMEM179* gene ($q$ = 1.16e-05 and $\Delta\beta$ = -0.26). CpG 2 is hypermethylated and resides at TSS1500 region on a densely populated CpG island of the *ISM1* gene (*q* = 8.89e-09, Δβ = 0.45). No methylation changes at gene promoter level was detected. Exposure to 100 µg/ml of SWCNT induced a single differential methylation of a CpG site by hypomethylation, however, the site could not be assigned to any gene. Gene promoter region of RNVU1-8 gene was identified to be significantly hypomethylated ($q$ = 2.32e-05, $\Delta\beta$ =-0.07).

The results remained subtle in order to relate CNT toxicity and no dose association was concluded. In order to improve the power of statistical analysis of the microarray ($N$=6), we have combined exposure doses (25 and 100 µg/ml) of each CNT exposure (MWCNT 25 and 100 µg/ml doses combined versus vehicle and control, SWCNT µg/ml doses combined versus vehicle and control).

## S. REFERENCES

1. NIST. Certificate of Analysis Standard Reference Material 2483 [Internet]. National Institute of Standards and Technology; 2011. Available from: https://www-s.nist.gov/srmors/certificates/2483.pdf

2. Rasmussen K, Mast J, De Temmerman P-J, Verleysen E, Waegeneers N, Van Steen F, et al. Multi-walled Carbon Nanotubes, NM-400, NM-401, NM-402, NM-403: Characterisation and Physico-Chemical Properties [Internet]. European commission; 2014. Report No.: 26796. Available from: https://ec.europa.eu/jrc/sites/default/files/mwcnt-online.pdf
